# ROS1 promotes low temperature-induced anthocyanin accumulation in apple by demethylating the promoter of anthocyanin-associated genes

**DOI:** 10.1093/hr/uhac007

**Published:** 2022-02-11

**Authors:** Lujia Yu, Yuying Sun, Xi Zhang, Mengchen Chen, Ting Wu, Jie Zhang, Yifan Xing, Ji Tian, Yuncong Yao

**Affiliations:** 1 Beijing Advanced Innovation Center for Tree Breeding by Molecular Design, Beijing University of Agriculture, Beijing, China; 2 Department of Plant Science and Technology, Beijing University of Agriculture, Beijing, China; 3 College of Horticulture, China Agricultural University, Beijing, China

## Abstract

Low temperature can affect the growth and development of plants through changes in DNA demethylation patterns. Another known effect of low temperature is the accumulation of anthocyanin pigments. However, it is not known whether the two phenomena are linked, specifically whether DNA demethylation participates in anthocyanin accumulation in response to low-temperature stress. The *ROS1* gene is involved in plant DNA demethylation and influences methylation levels in response to low-temperature stress. In this study, using RNA sequencing, we detected the transcription levels of *MdROS1*, as well as those of anthocyanin biosynthesis-related genes, correlate with the anthocyanin content in apple (*Malus domestica*), at low temperature. Genomic bisulfite sequencing showed that the methylation levels of the promoters of the anthocyanin-related genes *MdCHS*, *MdCHI*, *MdF3′H*, *MdANS*, *MdUFGT*, and *MdMYB10* decreased in apple leaves after low-temperature treatment. Similar expression and methylation results were found in apple fruit. Transiently silencing *MdROS1* in the leaves and fruit of apple cultivars inhibited the accumulation of anthocyanins and led to decreased expression of anthocyanin biosynthetic genes, and the opposite results were detected in *MdROS1*-overexpressing leaves and fruit. A promoter binding assay showed that the conserved RRD-DME domains of MdROS1 bind directly to the promoters of *MdF3′H* and *MdUFGT*. Taken together, these results suggest that ROS1 affects the anthocyanin biosynthetic pathway by decreasing the methylation level of anthocyanin-related gene promoters, thereby increasing their expression and increasing anthocyanin accumulation.

## Introduction

Anthocyanins are phenylpropanoid compounds that are ubiquitous in the tissues and organs of later-diverging land plants. These compounds have been shown to play multiple roles in environmental stress tolerance, in resistance to herbivores and pathogens, and as red, blue, and purple pigments that attract pollinators and seed dispersers [1–3]. Anthocyanins are also helpful in reducing several disease risks for humans as dietary supplements [4–7].

Biotic and abiotic factors such as light and temperature can affect anthocyanin synthesis [8, 9]. In apple, it is known that a lower temperature (12–17°C) promotes anthocyanin accumulation in fruit compared with a higher temperature (24–27°C) [10]. Furthermore, 7°C night temperature can also induce double the amount of anthocyanins compared with a 17°C night temperature [11]. Several studies have shown that low temperature (LT) promotes anthocyanin accumulation by inducing the transcription of anthocyanin biosynthesis-related genes [12–15]. LTs have been shown to facilitate anthocyanin biosynthesis by inducing the expression of several anthocyanin biosynthetic genes [16, 17], as well as members of the R2R3-MYB, basic helix-loop-helix (bHLH) and WD40 transcription factor families, which regulate anthocyanin biosynthesis under LT in crab apple (*Malus*) leaves [18]. It has also been shown that MdbHLH3, an LT-induced transcription factor, regulates LT-induced anthocyanin biosynthesis by binding to the promoters of the *MdDFR* and *MdUFGT* genes and *MdMYB1* to promote their expression in apple (*Malus domestica*) fruit [10]. In contrast, the MdMYB15L repressor binds to the promoter of *MdCBF2* and inhibits the expression of *MdCBF2*, thus decreasing the cold tolerance of apple at low temperature. This repressor also competitively binds with MdbHLH33 and reduces MdbHLH33-induced anthocyanin accumulation [19]. However, the upstream mechanism of the transcriptional regulation of LT-induced anthocyanin accumulation still needs to be examined.

LT affects plant development by coordinating gene expression at the transcriptional, posttranscriptional and posttranslational levels [20, 21]. DNA methylation and demethylation are important epigenetic modifications in plants, and they enhance the plasticity of the genome, allowing adaptations to environmental changes, as well as regulating growth and development [22, 23]. For example, LT can lead to changes in the level of DNA methylation. In maize (*Zea mays*), LT results in reduced methylation levels and increased expression of antiretroviral genes, which results in enhanced stress resistance and adaptability to LT stress [24]. In another study, it was reported that >60% of genes in the flower bud of peony (*Paeonia*) were methylated during dormancy, but 24 days of LT treatment ended the dormancy period, and the degree of methylation decreased by ~17%, consistent with DNA demethylation regulating LT-induced dormancy [25]. However, whether DNA demethylation can promote anthocyanin accumulation in response to LT stress is still unknown.

In plants, the DNA glycosylase DEMETER (DME) family members DME, DML2, DML3, and REPRESSOR OF SILENCING 1 (ROS1) carry out active DNA demethylation functions. All these DME/ROS1 proteins function in development. DME plays a vital role in seed development and endosperm gene imprinting [26–28] because *DME* is mainly expressed in the central cell of the female gametophyte, while *ROS1*, *DML2*, and *DML3* preferentially accumulate in vegetative tissues [29]. A forward genetic screen showed that a mutation of the *ROS1* gene enhanced the transcriptional silencing of many loci by DNA hypermethylation [22, 23]. *ROS1*-mediated DNA demethylation and gene regulation have been shown to control diverse processes, including antibacterial defense [30], seed dormancy [31], and the production of stomatal stem cells [32]. However, the cellular processes regulated by ROS1 have not been elucidated in detail.

Recent research has shown that massive expression of *AtROS1* from *Arabidopsis* in transgenic tobacco (*Nicotiana tabacum*) increases the demethylation levels of flavonoid biosynthetic genes during salt stress [33]. In peach (*Prunus persica*) flesh, DNA demethylation regulates temperature-dependent anthocyanin biosynthesis mainly by facilitating the expression of anthocyanin biosynthesis genes [34]. Studies have also shown that the demethylation of *DFR* and *Ruby* promoters was determined by DML1, and the expression of DML1 is induced by LT in highly pigmented areas of the fruits. The expression level of anthocyanin-related genes in blood orange fruit is related to epigenetic control mechanisms such as promoter methylation [35]. These results suggest that DNA demethylation may participate in LT-induced anthocyanin accumulation. Our recent study indicated that cold-responsive ROS1 protein positively correlated with anthocyanin accumulation in apple [36]. In this study, we tested the hypothesis that ROS1-mediated DNA demethylation is involved in LT-induced anthocyanin biosynthesis in apple leaves and fruit.

## Results

### Identification of the low-temperature-responsive gene *ROS1*

**Figure 1:**
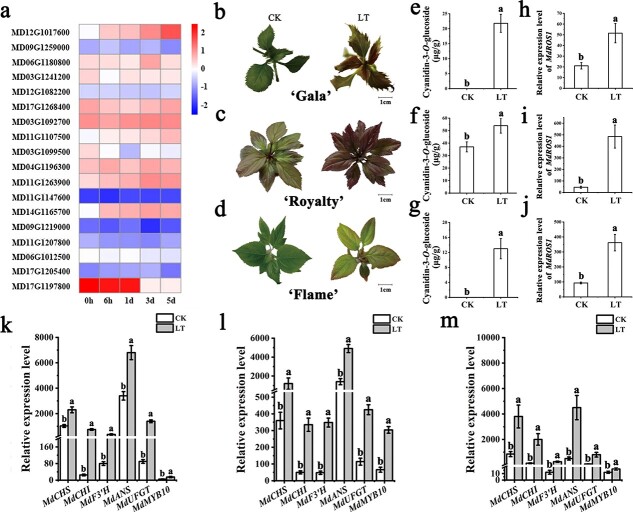
*ROS1* may participate in LT-induced anthocyanin accumulation in *Malus* plants. **a** Clustering heatmap of genes involved in DNA demethylation during LT treatment. **b**–**d** ‘Gala’, ‘Royalty’, and ‘Flame’ leaves under 23°C for 7 days were defined as control (CK). Scale bars = 1 cm. **e**–**g** Anthocyanin content in apple leaves following 7 days of LT treatment. **h**–**j** Expression level of *MdROS1* determined by qRT–PCR. **k**–**m** Expression levels of anthocyanin-related genes determined by qRT–PCR. Different letters above bars indicate significantly different values (*P* < .05) calculated using one-way ANOVA followed by Tukey’s multiple range test.

To investigate the function of genes involved in DNA demethylation during LT-induced anthocyanin accumulation, we searched transcriptome data from leaves of LT-treated apple (*M. domestica* ‘Gala’) plantlets [36] (Fig. 1a) and identified 18 homologous *Arabidopsis* DNA demethylation genes. The expression level of four of these genes increased with LT treatment, and expression analysis by quantitative reverse transcription PCR (qRT–PCR) showed that, of these four, MD12G1017600, a *ROS1* gene, had increasing expression and the highest transcript accumulation on Day 5 of treatment, so we targeted this gene for further analysis.

We compared the amino acid sequences of the *MdROS1* gene in different plants and found that they had high homology (Supplementary Fig. S1). MdROS1 is closely related to PbDME2 (*Pyrus bretschneideri*), PpROS1 (*P. persica*), PmROS1 (plum blossom), RcROS1 (*Rosa chinensis*) and FvROS1 (*Fragaria vesca* subsp. *vesca*) (Supplementary Fig. S2). To further determine whether this *MdROS1* gene responds to an LT signal, we measured its expression in the leaves of ‘Gala’ tissue culture seedlings after 16°C (LT) treatment for 7 days. As shown in Fig. 1b, a bright red color appeared in the leaves after this treatment. High-performance liquid chromatography (HPLC) analysis showed that anthocyanin levels (cyanidin-3-*O*-glucoside) increased from 0.0 to 22.30 μg/g after 7 days of LT treatment (Fig. 1e). Expression analysis showed that LT treatment significantly promoted the expression of *MdROS1*, and we also observed that anthocyanin biosynthesis genes [CHS (chalcone synthase), CHI (chalcone isomerase), flavonoid 3′-monooxygenase (F3′H), anthocyanidin synthase (ANS), and UDP-glucose:flavonoid 3-*O*-glucosyltransferase (UFGT)] and the regulatory gene *MdMYB10* showed increased expression (Fig. 1h and k).

We next performed LT treatment on the ever-red crab apple cultivar ‘Royalty’ and the evergreen crab apple cultivar ‘Flame’ (Fig. 1c–d), and we observed similarly increased expression of *ROS1* and anthocyanin biosynthesis and regulatory genes and anthocyanin accumulation under LT in these two cultivars (Fig. 1f–g, i–j and l–m), suggesting that *ROS1* participates in LT-induced anthocyanin accumulation in *Malus* plants.

### Changes in the expression of anthocyanin-related genes after *MdROS1* silencing in *Malus* leaves

**Figure 2:**
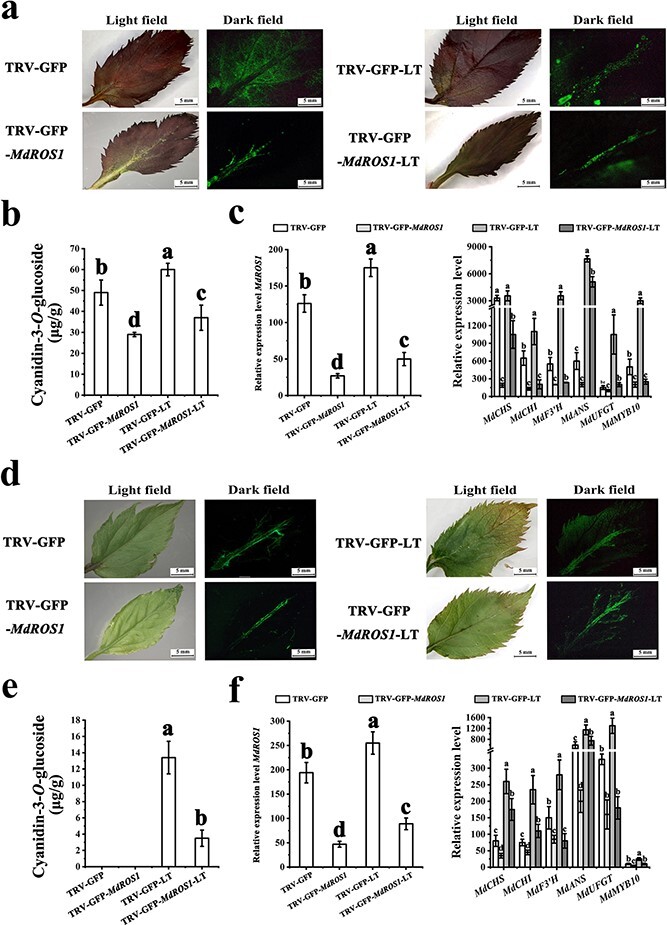
Transient silencing of *ROS1* in apple leaves. **a** Agroinfiltrated ‘Royalty’ leaves were photographed under UV illumination and normal light at 10 days post-infiltration. Scale bar = 1 cm. **b** Anthocyanin content in inoculated ‘Royalty’ leaves. **c** Expression of *MdROS1* and anthocyanin-related genes in infiltrated apple leaves was detected by qRT–PCR in infected ‘Royalty’ leaves. **d** Agroinfiltrated ‘Gala’ leaves were photographed under UV illumination and normal light at 10 days post-infiltration. **e** Anthocyanin content in inoculated ‘Gala’ leaves. Scale bar = 0.5 cm. **f** Expression of *MdROS1* and anthocyanin-related genes in inoculated ‘Gala’ leaves was determined using qRT–PCR. qRT–PCR and HPLC analyses were performed with three biological replicates. Error bars indicate the standard error of the mean (SE) of three replicate measurements. Different letters above the bars indicate significantly different values (*P* < .05), calculated using one-way ANOVA followed by Tukey’s multiple range test.

To further confirm the function of *MdROS1* in LT-induced anthocyanin accumulation, *Agrobacterium tumefaciens* cultures containing vectors for the expression of the green fluorescent protein (GFP) reporter alone (TRV-GFP) or GFP together with *MdROS1* silencing (TRV-GFP-*MdROS1*) were individually infiltrated into leaves of the ever-red crab apple cultivar ‘Royalty’ and the green-leaf apple cultivar ‘Gala’. Green fluorescence was observed in all infiltrated leaves. In ‘Royalty’, red leaves with suppressed *MdROS1* expression showed reduced accumulation of anthocyanins, resulting in green coloration in leaves infiltrated with the TRV-GFP-*MdROS1* vector after LT treatment (Fig. 2a). In ‘Gala’, there was no obvious variation between wild-type leaves and leaves that were *MdROS1*-silenced. We observed that LT treatment promoted anthocyanin accumulation and red coloration in control leaves, whereas the red color was weaker in leaves infiltrated with the TRV-GFP-*MdROS1* vector (Fig. 2d). Expression analysis showed that *MdROS1* was silenced (Fig. 2c and f), and HPLC analysis indicated significantly lower anthocyanin levels (cyanidin-3-*O*-glucoside) in the *MdROS1*-silenced leaves than in those of the control (Fig. 2b and e).

Quantitative qRT–PCR analysis suggested a significant decrease in the transcription of the anthocyanin regulatory gene *MdMYB10* and the anthocyanin biosynthetic genes *MdCHS*, *MdCHI*, *MdF3′H*, *MdANS*, and *MdUFGT* when *MdROS1* was silenced in two *Malus* cultivars under both normal temperature and LT (Fig. 2c and f) conditions. Next, we measured DNA methyltransferase (DNMT) activity in the *MdROS1*-silenced leaves and observed that activity increased substantially compared with control leaves under both normal and LT conditions (Supplementary Fig. S5).

### Overexpressing *MdROS1* in *Malus* leaves alters the expression of anthocyanin-related genes

**Figure 3:**
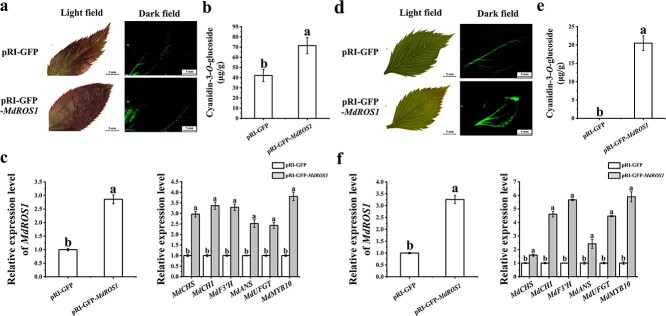
Transient overexpression of *ROS1* in apple leaves. **a** Agroinfiltrated ‘Royalty’ leaves were photographed under UV illumination and normal light at 10 days post-infiltration. Scale bar = 0.5 cm. **b** Anthocyanin content in inoculated ‘Royalty’ leaves. **c** Expression of *MdROS1* and anthocyanin-related genes was detected by qRT–PCR in infected ‘Royalty’ leaves. **d** Agroinfiltrated ‘Gala’ leaves were photographed under UV illumination and normal light at 10 days post-infiltration. Scale bar = 1 cm. **e** Anthocyanin content in inoculated ‘Gala’ leaves. **f** Expression of *MdROS1* and anthocyanin-related genes in inoculated ‘Gala’ leaves was determined using qRT–PCR. qRT–PCR and HPLC analyses were performed with three biological replicates. Error bars indicate the standard error of the mean (SE) of three replicate measurements. Different letters above the bars indicate significantly different values (*P* < .05), calculated using one-way ANOVA followed by Tukey’s multiple range test.

Deep red coloration was detected in *MdROS1*-overexpressing ‘Royalty’ leaves (Fig. 3a). The expression level of *MdROS1* confirmed that *MdROS1* was overexpressed (Fig. 3c and f). HPLC analysis also suggested significantly higher levels of cyanidin-3-*O*-glucoside in overexpressing leaves than in control ‘Gala’ leaves (Fig. 3b and e). qRT–PCR analysis suggested that the transcription levels of the anthocyanin regulatory gene *MdMYB10* and the anthocyanin biosynthetic genes *MdCHS*, *MdCHI*, *MdF3′H*, *MdANS*, and *MdUFGT* were significantly higher in the *MdROS1*-overexpressing leaves than in the control leaves (Fig. 3c and f). DNMT activity was suppressed in the *MdROS1*-overexpressing leaves compared with the control leaves (Supplementary Fig. S5).

### *MdROS1* is involved in low temperature-induced anthocyanin biosynthesis in apple fruit

**Figure 4:**
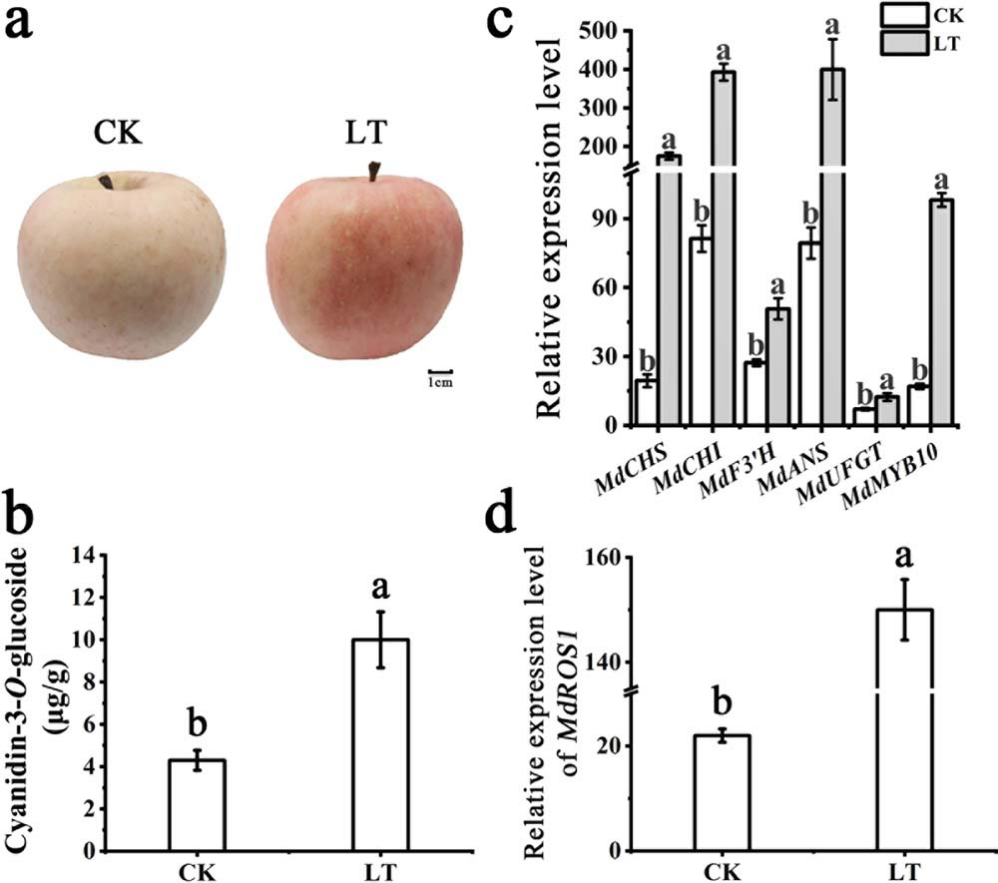
*ROS1* may participate in LT-induced anthocyanin accumulation in apple fruit. **a** ‘Red Fuji’ fruit under 23°C for 3 days were defined as control (CK). Scale bar = 1 cm. **b** Anthocyanin content in apple fruit following 3 days of LT treatment. **c** Expression level of anthocyanin-related genes determined by qRT–PCR. **d** Expression level of *MdROS1* determined by qRT–PCR. Different letters above the bars indicate significantly different values (*P* < .05) calculated using one-way ANOVA followed by Tukey’s multiple range test.

To further understand whether *MdROS1* is involved in apple fruit coloration after LT treatment, we stored debagged ‘Red Fuji’ fruit (140 days after bloom) at 16°C for 3 days. The results showed that the apple skin displayed significant anthocyanin accumulation after 3 days of LT treatment (Fig. 4a). HPLC analysis suggested that the anthocyanin content (cyanidin-3-*O*-glucoside) increased significantly after 3 days of LT treatment (Fig. 4b). qRT–PCR analysis of the LT-treated apple skins suggested that the transcription level of *MdROS1* was significantly upregulated by LT treatment and shared a similar trend with anthocyanin biosynthesis and regulatory genes (Fig. 4c and d). These results suggested that *MdROS1* may also play a crucial role in LT-induced anthocyanin accumulation in apple fruit.

### Changes in transcription of anthocyanin-related genes after *MdROS1* silencing in apple fruit

**Figure 5:**
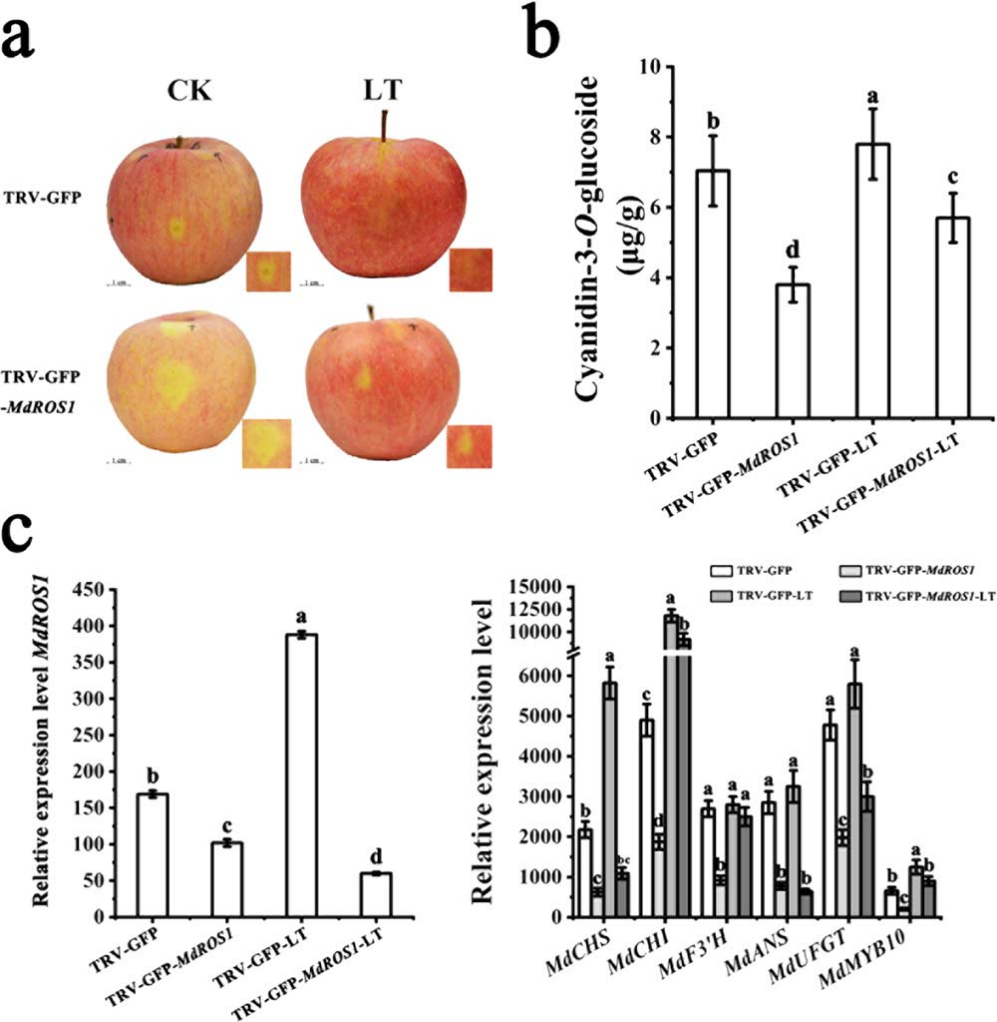
Transient silencing of *ROS1* in apple fruit. **a** Infiltrated apple fruits were visualized at 7 days post-infiltration. **b** Anthocyanin accumulation in inoculated apple fruit. (c) The transcription levels of *MdROS1* and anthocyanin biosynthesis genes in inoculated apple fruit were determined using qRT–PCR. qRT–PCR and HPLC analyses were performed with three biological replicates. Error bars indicate the standard error of the mean ± standard error of three replicate measurements. Different letters above the bars indicate significantly different values (*P* < .05) calculated using one-way ANOVA followed by Tukey’s multiple range test.

To further confirm the function of *MdROS1*, we also silenced its expression in apple fruit by agroinfiltration. Fruit that was transformed with TRV-GFP-*MdROS1* showed less anthocyanin accumulation at the injection sites, resulting in significant yellow coloration (Fig. 5a), while LT treatment led to increased accumulation of anthocyanins in control fruit, with a red coloration at the injection sites. HPLC analysis supported the phenotypic observations since anthocyanin levels (cyanidin-3-*O*-glucoside) in the *MdROS1*-silenced fruit were lower than those in the control fruit (Fig. 5b). Expression analysis confirmed that *MdROS1* was silenced (Fig. 5c). The expression levels of *MdMYB10* and the anthocyanin biosynthetic genes *MdCHS*, *MdCHI*, *MdF3′H*, *MdANS*, and *MdUFGT* were lower in *MdROS1*-silenced fruit. Under LT treatments, the *MdROS1*-silenced fruit showed lower expression of these genes than the control fruit (Fig. 5c). DNMT activity in the *MdROS1*-silenced fruit was much higher than that in the control fruit under both normal and LT conditions (Supplementary Fig. S5).

### Overexpressing *MdROS1* in apple fruit alters expression of anthocyanin-related genes

**Figure 6:**
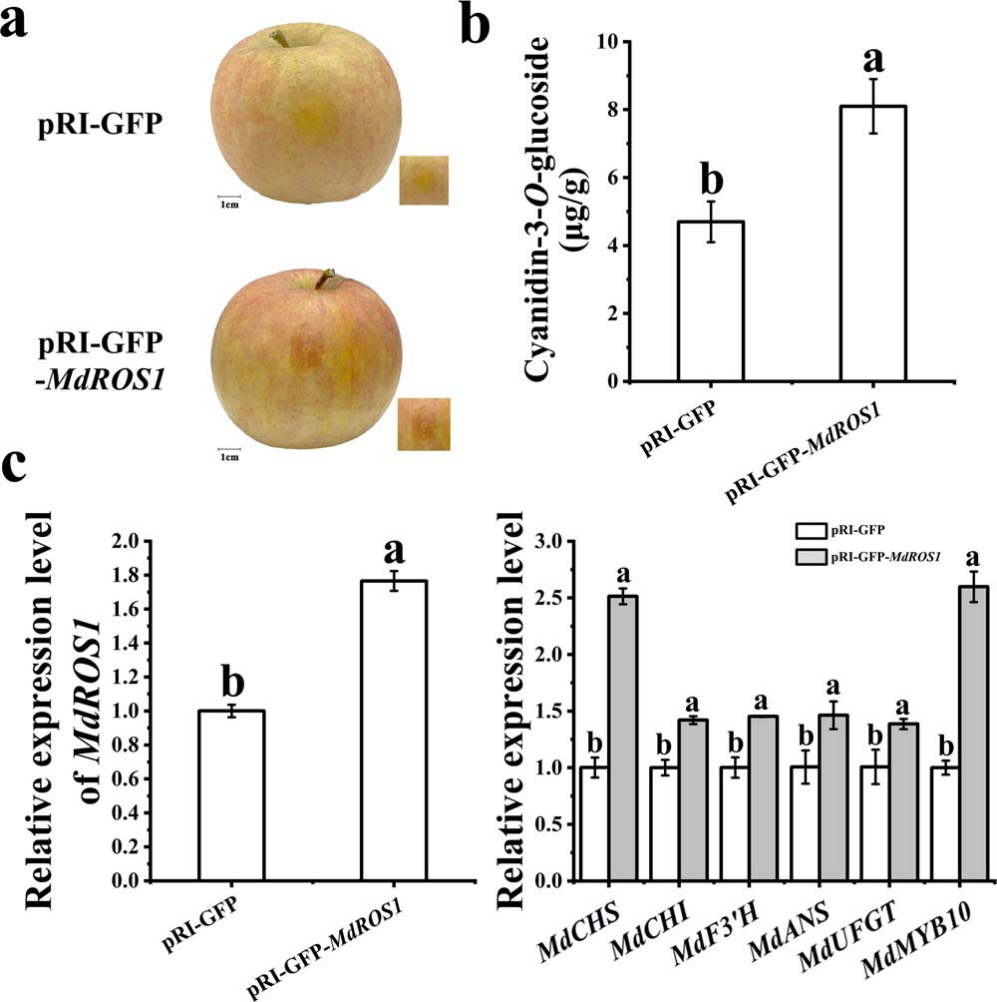
Transient overexpression of *ROS1* in apple fruit. **a** Infiltrated apple fruits were visualized at 3 days post-infiltration. **b** Anthocyanin accumulation in inoculated apple fruit. **c** Transcription levels of *MdROS1* and anthocyanin biosynthesis genes in inoculated apple fruit were determined using qRT–PCR. qRT–PCR and HPLC analyses were performed with three biological replicates. Error bars indicate the standard error of the mean ± standard error of three replicate measurements. Different letters above the bars indicate significantly different values (*P* < .05) calculated using one-way ANOVA followed by Tukey’s multiple range test.

To further test the function of *MdROS1*, the pRI-*MdROS1* vector was injected into the skin of apple fruit. As shown in Fig. 6a and b, overexpression of *MdROS1* promoted anthocyanin accumulation. The expression level of *MdROS1* was significantly higher in overexpressed fruit than in the control (Fig. 6c). Furthermore, the *MdROS1*-overexpressing fruit showed higher *MdMYB10*, *MdCHS*, *MdCHI*, *MdF3′H*, *MdANS*, and *MdUFGT* expression than the control fruit. DNMT activity in the *MdROS1*-overexpressing fruit was much lower than that in the control fruit (Supplementary Fig. S5). Taken together, these data suggest that *MdROS1* promotes LT-induced anthocyanin biosynthesis by regulating the expression of anthocyanin-related genes.

### DNA methylation levels in leaves and fruit after low-temperature induction of *ROS1*

**Figure 7:**
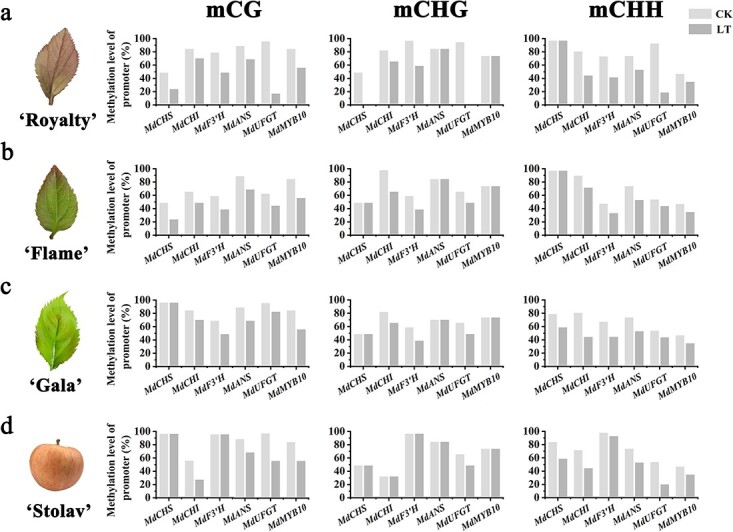
Detection of methylation levels. The methylation levels of the promoters of the anthocyanin regulatory gene *MdMYB10* and anthocyanin biosynthesis genes were detected by BSP in DNA from leaves and fruit. Fruit and leaves under 23°C for 3 days were defined as control (CK).

We next measured the methylation levels in the promoters of the anthocyanin regulatory gene *MdMYB10* and the anthocyanin biosynthesis genes by bisulfite-sequencing PCR (BSP) of genomic DNA from leaves and fruit. BSP analysis was performed as previously described [37]. Primers were designed by MethPrimer (http://www.urogene.org/methprimer/) and were placed as described in Supplementary Fig. S3. In ‘Royalty’ leaves, BSP analysis indicated that the methylation levels decreased under LT, most notably in *MdF3′H* and *MdUFGT* (Fig. 7a). In ‘Flame’ leaves, methylation mainly occurred in the CHH sequence and to a lesser extent in the CHG and GC sequences. Decreased methylation was also detected in ‘Flame’ and ‘Gala’ leaves (Fig. 7b and c). We also observed that methylation also occurred in the fruit peels of ‘Stolav’ under LT, mainly in the CHH sequence and especially in *MdUFGT* (Fig. 7d).

### MdROS1 protein binds to the promoters of anthocyanin biosynthetic genes

**Figure 8:**
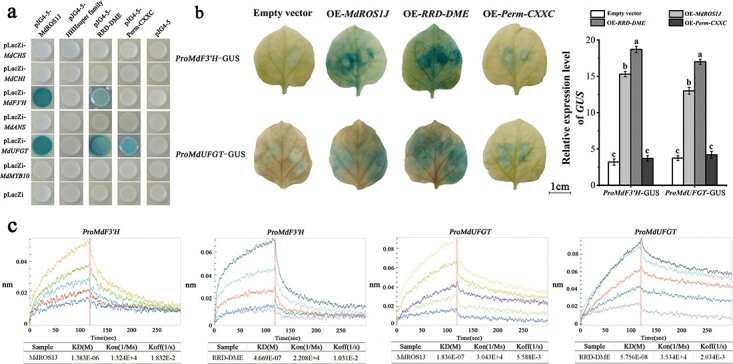
The MdROS1 protein binds to the promoters of anthocyanin-related genes. **a** Yeast one-hybrid assay indicating that the perm-CXXC and RRD-DME domains and MdROS1J protein bind directly to the promoters of *MdF3′H* and *MdUFGT*. **b** Transient transactivation assay in tobacco leaves using the *β-glucuronidase* (*GUS*) reporter gene. GUS staining shows that OE-MdROS1J and OE-RRD-DME activate *MdF3′H* and *MdUFGT* promoter activity and accumulate GUS protein in tobacco leaves. **c** A biolayer interferometry (BLI) assay was used to quantify the binding affinities of the RRD-DME domains and MdROS1J protein to the *MdF3′H* and *MdUFGT* promoters. Protein concentrations were 1250 nM (upper lines) and 78.1 nM (lower lines). Different letters above the bars indicate significantly different values (*P* < .05) calculated using one-way ANOVA followed by Tukey’s multiple range test.

The results of the *MdROS1* silencing and BSP analyses indicated that the *ROS1*-mediated DNA demethylation pathway is involved in LT-induced anthocyanin accumulation. To further confirm the function of MdROS1 binding to the promoters of anthocyanin-related genes, a yeast one-hybrid (Y1H) assay was conducted. The main domains, helix-hairpin-helix motif (HHH superfamily, 3769–4285 bp), permuted single zf-CXXC (Perm-CXXC, 4957–5052 bp), and RNA recognition motif-DME (RRD-DME, 5059–5365 bp), were inserted into the pJG4-5 vector. These three domains were joined with the MdROS1J sequence and inserted into the pJG4-5 vector (Supplementary Fig. S4). The results showed that MdROS1J and the RRD-DME domain bound to the promoters of *MdF3′H* and *MdUFGT* and that the perm-CXXC domain bound the promoter *MdUFGT*. This suggested that the anthocyanin biosynthetic genes *MdF3′H* and *MdUFGT* might be MdROS1 target genes (Fig. 8a). To provide more evidence for the interaction, we performed a β-glucuronidase (GUS) staining assay using the pGFPGUSPLUS vector containing the MdROS1J or RRD-DME sequence in tobacco. The results showed that tobacco expressing both MdROS1J and *MdF3′H*, RRD-DME and *MdF3′H*, MdROS1J and *MdUFGT*, and RRD-DME and *MdUFGT* were dyed blue (Fig. 8b), indicating that the RRD-DME and MdROS1J sequences had higher affinity for the *MdF3′H* and *MdUFGT* promoters. Furthermore, we cut the promoter into three segments, performed Y1H assays (Supplementary Fig. S6) and conducted a biolayer interferometry (BLI) assay to quantify the binding affinities of the RRD-DME and MdROS1J protein sequences to the promoters of *MdF3′H* and *MdUFGT*. The results showed that RRD-DME and MdROS1J had a significant interaction with the *MdUFGT* promoter. The kinetic values indicated that RRD-DME had a higher affinity for the *MdUFGT* promoter than did MdROS1J (Fig. 8c).

## Discussion

LT promotes anthocyanin accumulation in *Malus* plants [38], and anthocyanins play an important role in plant adaptation to changes in environmental conditions [1–3]. Here, we demonstrate that ROS1, a DNA demethylation protein, is a positive regulator of cold tolerance and anthocyanin biosynthesis in apple leaves and fruit. In addition, ROS1 was confirmed to bind directly to downstream gene promoters to regulate their methylation levels.

### Under low-temperature stress, ROS1 participates in the formation of anthocyanins

ROS1-mediated DNA demethylation has been studied extensively in plants. For example, the DNA demethylase ROS1 can regulate seed dormancy by controlling imprinted gene transcription in the endosperm in *Arabidopsis* [31]. The silencing of *EPIDERMAL PATTERNING FACTOR 2* (*EPF2*) in *Arabidopsis* with mutated *ROS1* resulted in the overproduction of stomatal lineage cells [32]. In rice, defects in male and female gametogenesis were observed on knocking out the *OsROS1* mutant [39]. Moreover, *OsROS1* can promote aleurone formation by reducing the expression levels of two putative transcription factor genes, *RISBZ1* (rice seed b-Zipper 1) and *RPBF* (rice prolamin box binding factor) [40]. ROS1 also plays an important role during plant defense against pathogens. The expression of several immunity-related genes was enhanced by demethylating the promoters of these genes by ROS1 to restrict attack by the bacterium *Pseudomonas syringae* [30]. These studies suggested that ROS1 participates in various physiological activities during plant growth and development.

We also noticed that several studies showed that DNA demethylation and methylation can affect flavonoid biosynthesis during cold treatment [34, 35]. For example, demethylation of the *Artemisia annua AaPAL1* promoter strongly correlated with anthocyanin accumulation [46], and the expression level of the DNA demethylase *ROS1* gene peaked at the pink stage of strawberry ripening [50]. In our present study, we found that LT treatment induced the expression of *ROS1*, accompanied by anthocyanin accumulation, as well as anthocyanin-related gene expression. Functional assays also suggested that the expression variation in *MdROS1* can alter the coloration of *Malus* leaves and fruit, suggesting that *ROS1* may be an anthocyanin regulatory gene at the transcription level, promoting anthocyanin accumulation during LT stress.

### ROS1-mediated DNA demethylation is a supplementary mechanism for LT-induced anthocyanin accumulation

Anthocyanin synthesis is induced by various environmental factors, such as light, LT, and salinity [41–43]. Several studies have shown that LT leads to an increase in anthocyanin biosynthesis mainly by promoting the transcription of relevant transcription factors [10, 12, 15, 44]. In *Arabidopsis thaliana*, MYB75, MYB90, MYB113, and MYB114 contribute to LT-induced anthocyanin accumulation [45–47]. BoPAP1, a MYB transcription factor, has been shown to increase the expression level of anthocyanin biosynthesis genes and promote coloration in purple kale (*Brassica oleracea*) during LT exposure [44]. An apple MYB transcription factor, MdMYB15L, was found to inhibit anthocyanin accumulation by competitively interacting with MdbHLH33 under cold treatment [19]. Finally, MdSIZ1 was identified to regulate anthocyanin accumulation by sumoylating MdMYB1 in response to LT in apple [48].

Another recent study showed that massive expression of *AtROS1* in tobacco leads to a reduction in the methylation of the *CHS*, *CHI*, *F3H*, *FLS*, *DFR*, and *ANS* promoters and induces their expression, resulting in an elevated flavonoid content [33]. Moreover, lower methylation levels in the promoters of anthocyanin biosynthesis genes and regulatory genes were positively related to the higher expression levels of these genes in LT-treated peach fruit flesh [34]. The methylation levels of both the *DFR* and *Ruby* promoter regions were strongly decreased in the area of high anthocyanin accumulation during cold storage. This report suggested that anthocyanin accumulation in blood orange fruit is related to epigenetic control mechanisms such as promoter methylation [35]. Therefore, we deduced that DNA demethylation in anthocyanin biosynthesis gene promoters may play an important role in regulating LT-induced anthocyanin accumulation in *Malus* plants. Our results showed that the methylation level of anthocyanin biosynthesis gene promoters was decreased in LT-treated *Malus* leaves or fruit, and the expression level of anthocyanin biosynthesis genes was significantly changed with *ROS1* overexpression or silencing, which suggested that LT can induce the demethylation of anthocyanin biosynthesis genes by *ROS1* and promote anthocyanin accumulation by increasing the expression of related genes. Furthermore, we hypothesized that ROS1-mediated demethylation of anthocyanin biosynthesis gene promoters may be a coordinated regulatory mechanism, with transcription factors controlling anthocyanin-related gene expression and regulating anthocyanin accumulation under LT.

### The RRD-DME domain of MdROS1 interacts directly with the promoter of *MdUFGT*

High levels of *AtROS1* expression in tobacco plants affect the methylation status of flavonoid biosynthesis genes and antioxidant-related gene promoters. *A. thaliana ros1* mutants have been reported to have hypermethylated genes/transgenes, resulting in transcriptional gene silencing [22]. *NtGPDL*-like genes were shown to be demethylated under abiotic stress conditions [33]. However, these studies were limited to epigenetic analysis and did not show how ROS1 interacts with the promoters of target genes.

A previous study showed that *Arabidopsis* anti-silencing factor SUVH1 (Su(var)3-9 homolog) is required for the transcription of promoter-methylated genes without altering DNA methylation [51]. In a ChIp-seq (chromatin immunoprecipitation combined with sequencing) assay SUVH1 was significantly enriched in the promoters of methylated genes, which suggested that SUVH1 can directly bind to these promoters [51]. These results suggest that MdROS1 may have the same mechanism when regulating DNA demethylation.

Our results showed that *MdROS1* can bind to the promoters of anthocyanin biosynthetic genes to participate in anthocyanin accumulation. MdROS1 has three structures in the conserved domain area: an HHH superfamily domain, a perm-CXXC domain, and an RRD-DME domain. The RRD-DME domain is an important structural domain in plant DNA glycosylases and participates in demethylation [52, 53]. The perm-CXXC domain has been shown to bind to non-methyl sites [53], and the HHH superfamily domain has DNA binding properties [54, 55]. Y1H assays showed that MdROS1J (the combined HHH motif, perm-CXXC domain, and RRD-DME domain) and the RRD-DME domain interacted with the promoters of *MdF3′H* and *MdUFGT*, and GUS staining assays and BLI further confirmed that the MdROS1J and RRD-DME domains interacted with the promoters of *MdF3′H* and *MdUFGT*.

We deduce that MdROS1-mediated DNA demethylation promotes anthocyanin biosynthesis in *Malus* plants by binding to the promoters of *MdF3′H* and *MdUFGT* under LT conditions and that the RRD-DME domain of MdROS1 is necessary for interaction with the target gene.

Furthermore, ROS1, DME, DML1, DML2, and DML3 belong to the 5-mC DNA glycosylase family of proteins and are responsible for initiating active DNA demethylation in *Arabidopsis* [22, 52, 56]. Moreover, the ROS1 loss-of-function mutant showed no obvious developmental defects, and we predict that these five 5-mC DNA glycosylase family proteins are functional complements in plants. In our results, we noticed that the methylation levels of anthocyanin-related genes, including *MdMYB10*, were reduced. Furthermore, Y1H, electrophoretic mobility shift assay (EMSA), and BLI assays suggested that *MdF3′H* and *MdUFGT* are direct targets of MdROS1. Therefore, we deduced that the DME, DML1, DML2, and DML3 proteins may also participate in the LT-induced anthocyanin biosynthesis process, and we will elucidate this hypothesis in our future study.

### DNA methylation and demethylation are dynamic processes

The biological processes of phosphorylation and acetylation are reversible, and the methylation of histones at lysine residues is also reversible [57]. During ROS1-mediated DNA demethylation, METHYL-CpG-BINDING DOMAIN 7 (MBD7) interacts with Increased DNA Methylation 2 (IDM2) and Increased DNA Methylation 3 (IDM3) to recruit Increased DNA Methylation 1 (IDM1) for histone acetylation and provides the proper chromatin environment for ROS1 function [58]. ROS1 antagonizes RNA-directed DNA methylation (RdDM) to avoid DNA hypermethylation at specific loci, and the expression of ROS1 decreases in all known RdDM mutants, indicating that *ROS1* expression is influenced by DNA methylation and demethylation pathways [49, 59, 60]. Studies have also shown that ROS1 expression is promoted by DNA methylation and antagonized by DNA demethylation [61], and ROS1 expression is also inhibited in RdDM silencing and in *met1* mutants [61]. Moreover, there is a cyclically reversible dynamism between DNA methylation and DNA demethylation during the temperature seasonality response in perennial American ginseng [62], which suggests that DNA methylation and demethylation are dynamic processes in plants.

The methylation levels of the *MdMYB10* promoter were related to the different apple peel color patterns [63]. Moreover, the expression of *MdMYB1* is mainly regulated by the CHH methylation levels in the MR3 region (−1246 bp to −780 bp) of the *MdMYB1* promoter. These results suggested that the expression of MYB1 is mainly controlled by DNA methylation [64]. In our study, BSP analysis suggested that the methylation level of the anthocyanin regulatory gene *MdMYB10*, as well as anthocyanin biosynthetic genes, decreased under LT. Therefore, we deduced that LT promoted the expression of *ROS1,* enhanced the demethylation of anthocyanin-related genes, and induced anthocyanin accumulation. This suggests that methylation and demethylation synergistically control the transcription of *MdMYB10* and anthocyanin biosynthetic genes in response to temperature variation.

In conclusion, this study indicates that ROS1 promotes anthocyanin accumulation under LT conditions by binding directly to the promoters of anthocyanin-related genes, resulting in dynamic methylation and demethylation. Moreover, ROS1-mediated DNA demethylation is a supplementary mechanism for LT-induced anthocyanin accumulation.

## Materials and methods

### Plant materials

The experimental materials used in this study were *M. domestica* ‘Gala’, ‘Red Fuji’, ‘Stolav’, *Malus* cv. ‘Royalty’, and *Malus* cv. ‘Flame’. The explants were taken from the annual branches in the apple germplasm resource garden of Beijing Agricultural University before germination in the spring, and were cultured in Murashige and Skoog (MS) medium. The culture temperature was 23–26°C, the relative humidity was controlled at 60–70%, the light duration was 16 hours/8 hours, and the light intensity was 10 000 lux. Bagged ‘Stolav’ fruit were collected 140 days after blooming. We used three trees with similar growth conditions and collected fruit samples from annual branches growing in the southeast direction. Fruit skins were collected by peeling to generate samples with <1 mm of cortical tissue.

### RNA extraction and qRT–PCR analysis

RNA samples were extracted from apple peels and crab apple leaves using an RNA Extraction Kit (Biomed, Beijing, China). SYBR Green qPCR Mix (TaKaRa, Ohtsu, Japan) and a Bio-Rad CFX96 Real-Time PCR System (BIO-RAD, USA) were used to analyze the expression of related genes as described before [18]. The 2^(−∆∆Ct)^ method [65] was used to calculate transcription levels.

### Measurement of methylation levels

Genomic DNA (1.0 μg) was obtained using the DNA Bisulfite Extraction Kit (Aidlab, Beijing, China) to conduct bisulfite treatment. The Methylation-Specific PCR Kit (TIANGEN BIOTECH, Beijing, China) was used for PCR analysis. The methylation level was calculated after sequencing 8–12 times .

### Yeast one-hybrid assays

As the effector constructs, the open reading frame of MdROS1 and the HHH superfamily, perm-CXXC, and RRM-DME structural domains were separately cloned into pJG4-5 vector (Clontech, Palo Alto, CA, USA) under the galactokinase 1 (GAL1) promoter. The *MdCHS*, *MdCHI*, *MdF3′H*, *MdANS*, *MdUFGT*, and *MdMYB10* promoter sequences were inserted in the pLacZi vector. The EGY48 yeast (*Saccharomyces cerevisiae*) strain was used to conduct the Y1H assay. All transformation and screenings were performed three times.

### Transient expression in tobacco and β-glucuronidase staining assays

Transient overexpression of MdROS1J, RRD-DME and Perm-CXXC in tobacco (*Nicotiana benthamiana*) leaves was performed using the pBI121 vector containing the MdROS1J, RRD-DME and Perm-CXXC sequence, using a previously described protocol [66]. The *MdUFGT and MdF3’H* promoter was cloned into the pBI101 vector. Transient expression assays in *N. benthamiana* plants were carried out as previously described [67]. GUS staining was performed as previously described [68]. All experiments were carried out with three biological replicates.

### Biolayer interferometry assays

GST-MdROS1J and GST-RRD-DME solution (20 μg/ml) diluted with 10 mM sodium acetate pH 4.5 was detected with sensors for 10 min to analysis the binding ability of MdROS1 with target promoters. Promoter sequences were diluted to five different concentrations in BLI buffer [0.01 M PBS, pH 7.4, 0.005 % (v/v) Tween 20] to serve as analyte samples [69].

### HPLC analysis

Apple leaf and fruit peel samples (0.8–1.0 g fresh weight) were ground in 10 ml extraction solution (methanol:water:formic acid:trifluoroacetic acid, 70:27:2:1) and incubated at 4°C in the dark for 72 hours [70]. The supernatant was passed through filter paper and then through a 0.22-μm Millipore™ filter (Billerica, MA, USA). An HPLC instrument (Agilent 1100, Agilent Technologies Inc., USA) with a 150-mm column was used for determination of anthocyanin content. When the retention time was ~5.9 minutes, the peak represented cyanidin-3-*O*-glucoside [3, 70, 71]. The peak area (*x*) was used to calculate anthocyanin content by the following formula:

Anthocyanin content = (*R* × *x* + *a*) · *v*/*m*

where *R* and *a* are the coefficients of the standard curve as measured with cyanidin-3-*O*-glucoside standard samples, *x* is the peak area, *v* is the volume of extraction solution, and *m* is the weight of samples.

### Transient expression assays in apple plantlets and apple fruit

The *MdROS1* (434 bp) fragment was cloned into pTRV2 vector at the XbaI and KpnI sites for a silencing assay [72]. The full length of *MdROS1* was cloned into a modified pRI101-eGFP vector at the NdeI and BamHI sites for overexpression analysis. The infiltration protocol and culture methods for transient expression assays in crab apple plantlets and apple fruit were as previously described [18, 73]. All samples were analyzed in at least three biological replicates.

## Supplementary Material

Web_Material_uhac007

## Data Availability

RNA-sequencing data in this study have been deposited in the NCBI Bioproject database under accession number (PRJNA732234).
