# Supplementary material for: ROS1 promotes low temperature-induced anthocyanin accumulation in apple by demethylating the promoter of anthocyanin-associated genes
Source: Hortic Res. 2022 Feb 11;9:uhac007. doi: 10.1093/hr/uhac007 (PMC9123231; doi:10.1093/hr/uhac007)
Supplement: Web_Material_uhac007 [file web_material_uhac007.zip › Supplemetal Figures.docx]

**Supplemental Figures**

**
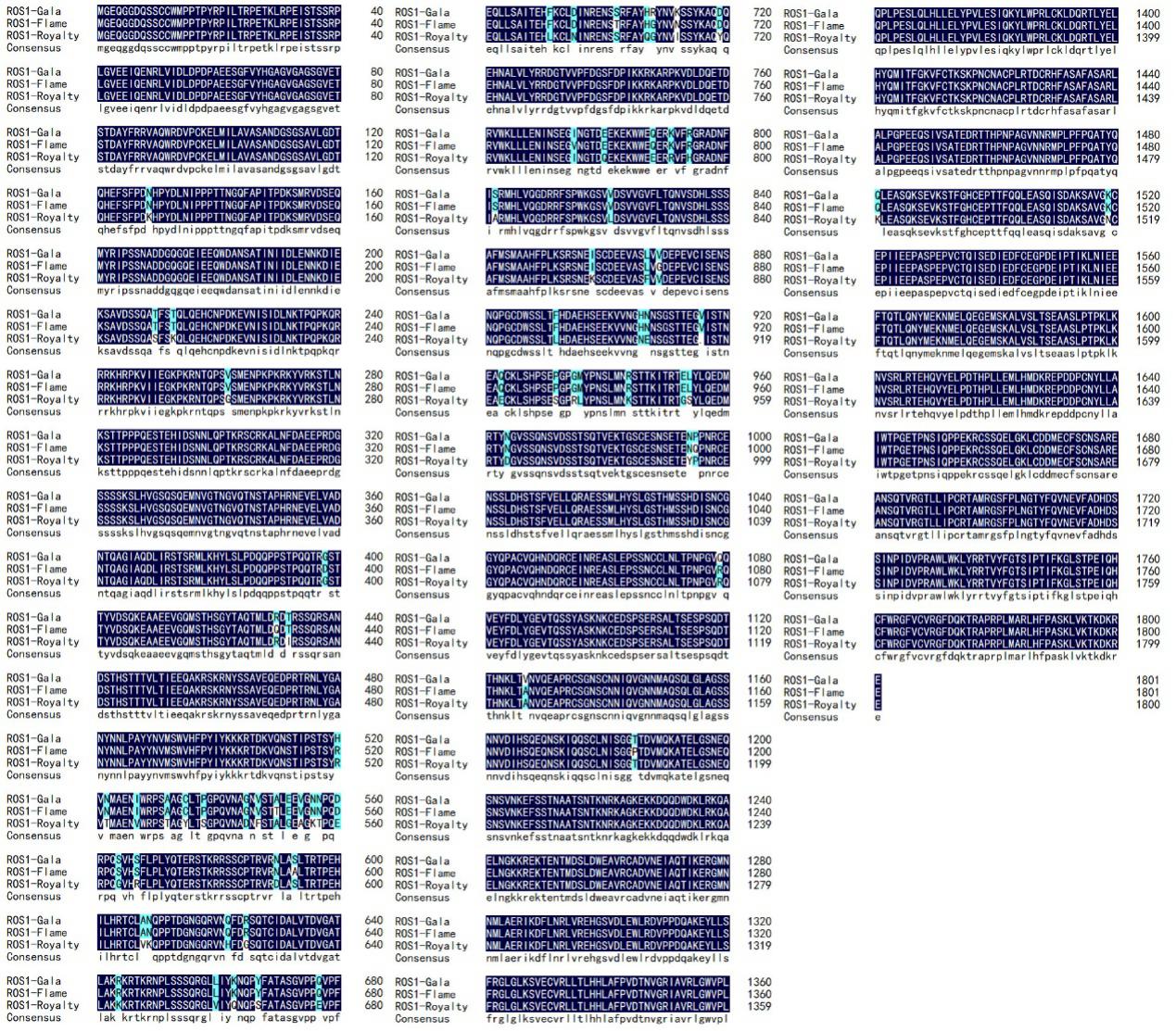
**

**Supplementary Fig. S1.** ROS1 amino acid sequence alignment in ‘Gala’, ‘Flame’ and ‘Royalty’. The full length was 1801 a.a., and the similarity of ROS1 in different experimental materials was 98.67%, indicating that the ROS1 amino acid sequence was conserved.

**
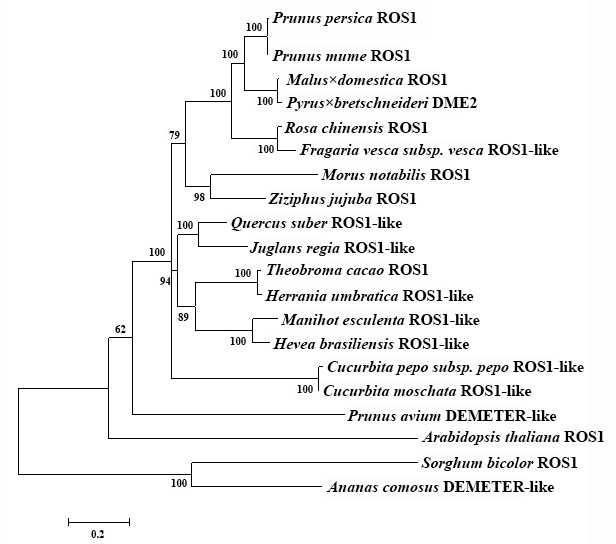
**

**Supplementary Fig. S2.** Phylogenetic analysis of *MdROS1* from various species. GenBank accession numbers are as follows: *Prunus persica* (XM_020569212.1), *Prunus mume* (XM_008243074.2), *Malus x domestica* (XM_008388713.2), *Pyrus x bretschneideri* (XM_009367047.2), *Rosa chinensis* (XM_024333589.1), *Fragaria vesca subsp. Vesca* (XM_011468495.1), *Quercus suber* (XM_024072554.1), *Juglans regia* (XM_018956827.1), *Theobroma cacao* (XM_018121526.1), *Herrania umbratica* (XM_021434325.1), *Manihot esculent* (XM_021766314.1), *Hevea brasiliensis* (XM_021823102.1), *Morus notabilis* (XM_024165883.1), *Ziziphus jujube* (XM_016043213.2), *Cucurbita pepo subsp. Pepo* (XM_023684424.1), *Cucurbita moschata* (XM_023084190.1), *Prunus avium* (XM_021977923.1), *Arabidopsis thaliana* (AY286009.1), *Sorghum bicolor* (XM_021460038.1) and *Ananas comosus* (XM_020245472.1).

**
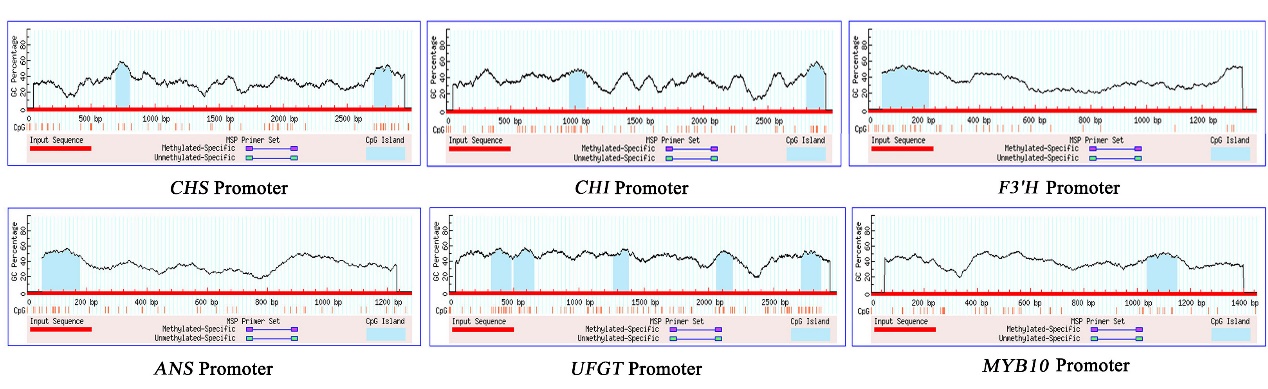
**

**Supplementary Fig. S3.** DNA methylation sites in promoters of anthocyanin biosynthesis genes (*MdCHS*, *MdCHI*, *MdF3’H*, *MdANS*, *MdUFGT*) and *MdMYB10*.

**
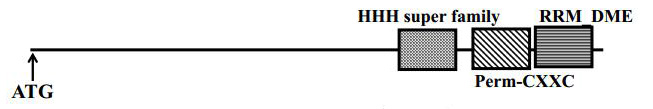
**

**Supplementary Fig. S4.** Structural analysis of *MdROS1* conserved domains (CDs). There are three domains in the *MdROS1* CD area: a helix-hairpin-helix motif (HHH super family, 3769 bp-4285 bp), a permuted single zf-CXXC domain (perm-CXXC, 4957 bp-5052 bp), and a RNA recognition motif-DME domain (RRD-DME, 5059 bp-5365 bp).


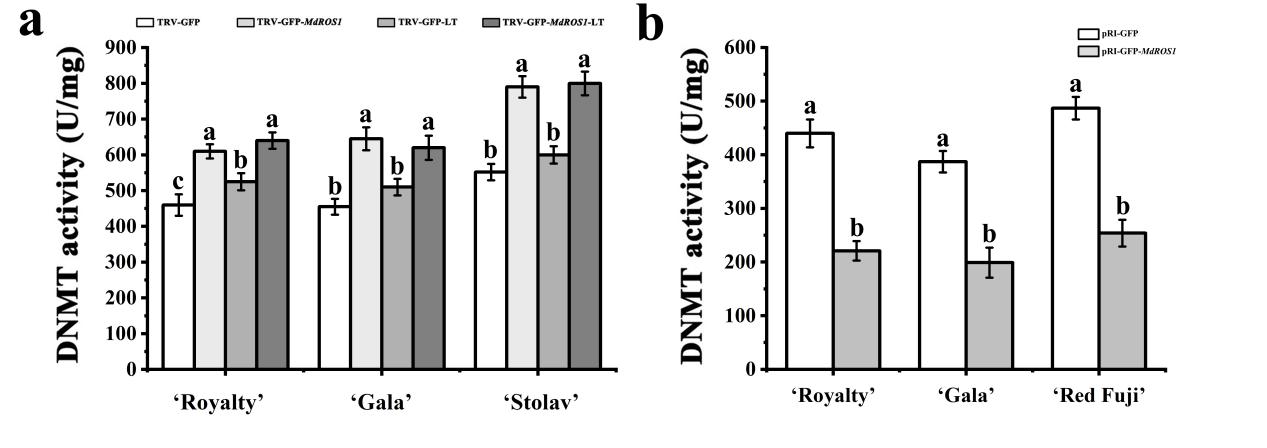


**Supplementary Fig. S5.** (a) The DNMT activity in *MdROS1* silenced leaves and fruits. (b) The DNMT activity in the *MdROS1*-overexpressed leaves and fruits. Different letters above the bars indicate significantly different values (P < 0.05) calculated using one-way analysis of variance (ANOVA) followed by a Tukey’s multiple range test.


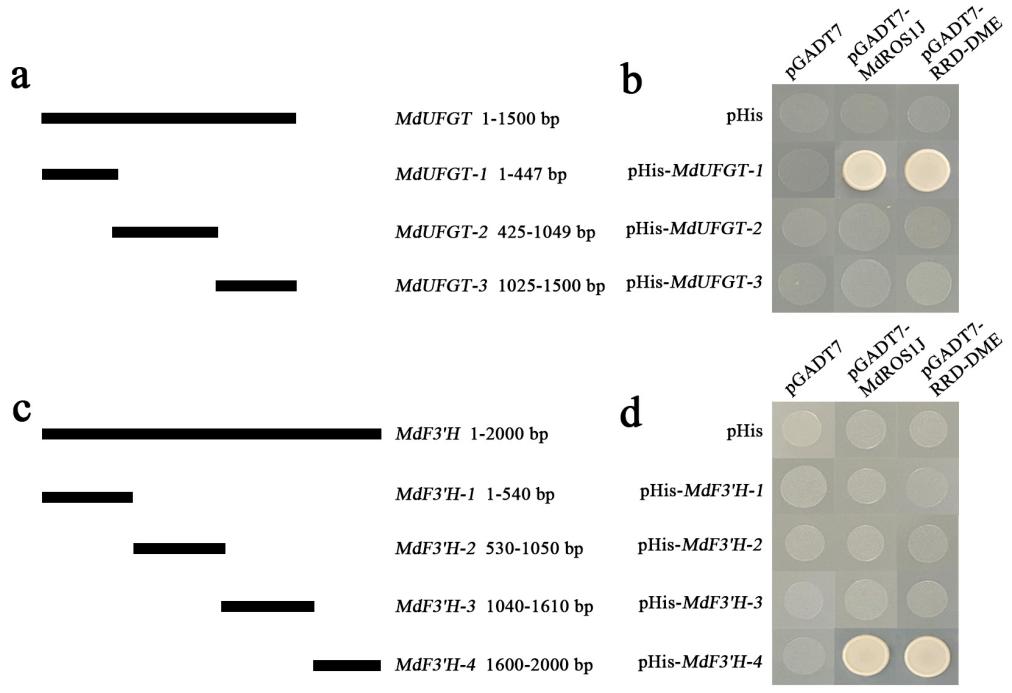


**Supplementary Fig. S6.** Yeast one-hybrid assay indicating that the RRD-DME domains and MdROS1J protein binds directly to the promoters of *MdF3’H-4* (1600 bp-2000 bp) and *MdUFGT-1* (1 bp-447 bp).
