# Supplementary material for: ROS1 promotes low temperature-induced anthocyanin accumulation in apple by demethylating the promoter of anthocyanin-associated genes
Source: Hortic Res. 2022 Feb 11;9:uhac007. doi: 10.1093/hr/uhac007 (PMC9123231; doi:10.1093/hr/uhac007)
Supplement: Web_Material_uhac007 [file web_material_uhac007.zip › Supplemetal Figures.pdf]

Supplemental Figures

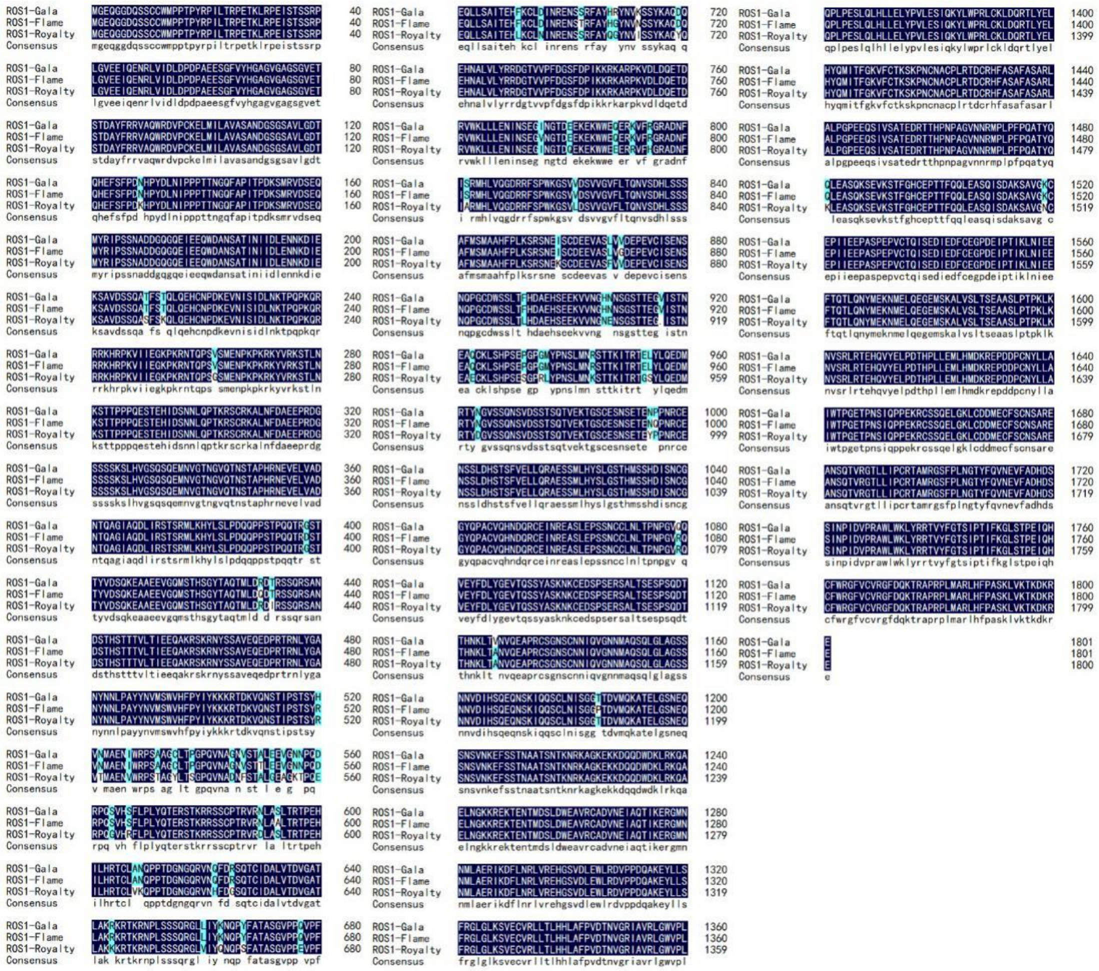

Supplementary Fig. S1. ROS1 amino acid sequence alignment in ‘Gala’, ‘Flame’ and ‘Royalty’. The full length was 1801 a.a., and the similarity of ROS1 in different experimental materials was 98.67%, indicating that the ROS1 amino acid sequence was conserved.

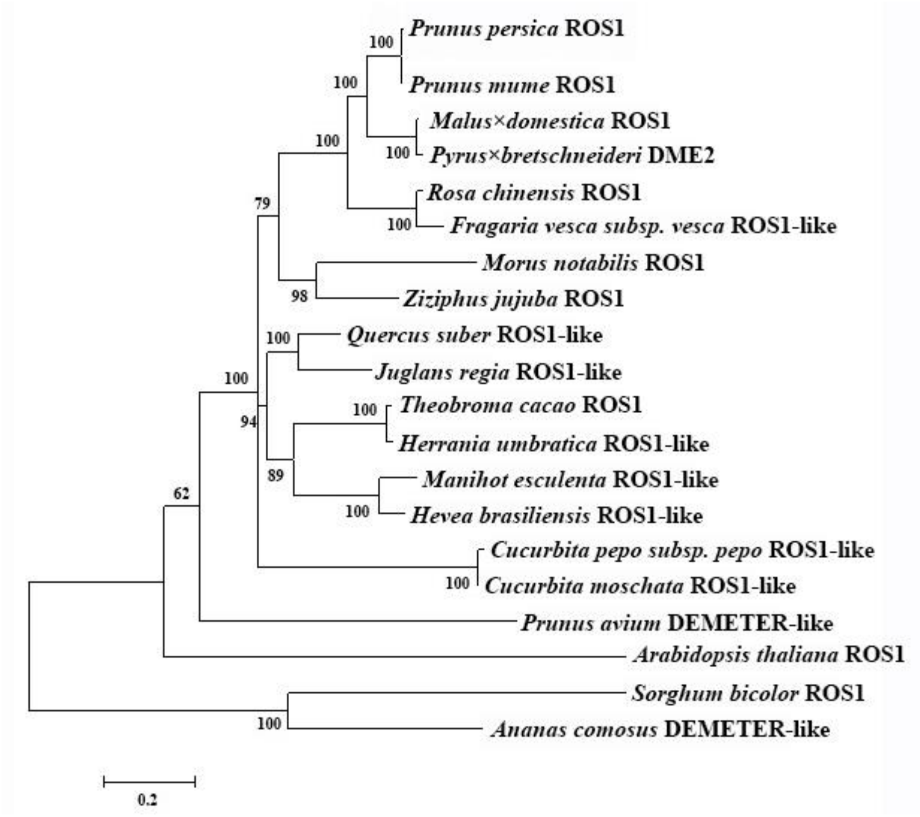

Supplementary Fig. S2. Phylogenetic analysis of *MdROS1* from various species. GenBank accession numbers are as follows: *Prunus persica* (XM_020569212.1), *Prunus mume* (XM_008243074.2), *Malus x domestica* (XM_008388713.2), *Pyrus x bretschneideri* (XM_009367047.2), *Rosa chinensis* (XM_024333589.1), *Fragaria vesca subsp. Vesca* (XM_011468495.1), *Quercus suber* (XM_024072554.1), *Juglans regia* (XM_018956827.1), *Theobroma cacao* (XM_018121526.1), *Herrania umbratica* (XM_021434325.1), *Manihot esculent* (XM_021766314.1), *Hevea brasiliensis* (XM_021823102.1), *Morus notabilis* (XM_024165883.1), *Ziziphus jujube* (XM_016043213.2), *Cucurbita pepo subsp. Pepo* (XM_023684424.1), *Cucurbita moschata* (XM_023084190.1), *Prunus avium* (XM_021977923.1), *Arabidopsis thaliana* (AY286009.1), *Sorghum bicolor* (XM_021460038.1) and *Ananas comosus* (XM_020245472.1).

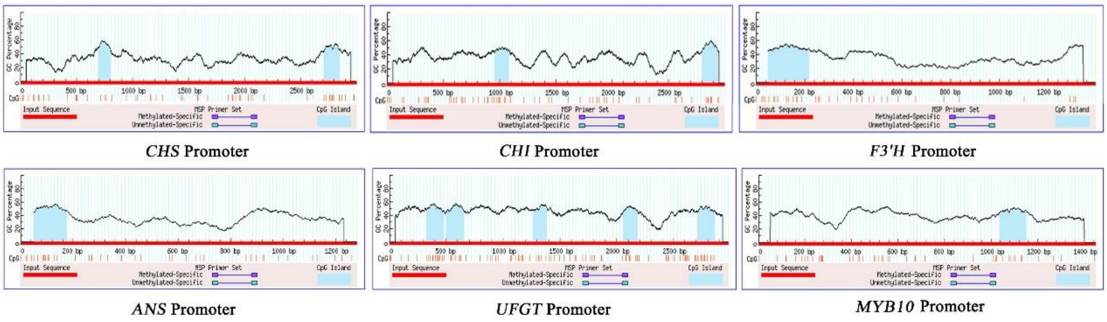

Supplementary Fig. S3. DNA methylation sites in promoters of anthocyanin biosynthesis genes (*MdCHS*, *MdCHI*, *MdF3'H*, *MdANS*, *MdUFGT*) and *MdMYB10*.

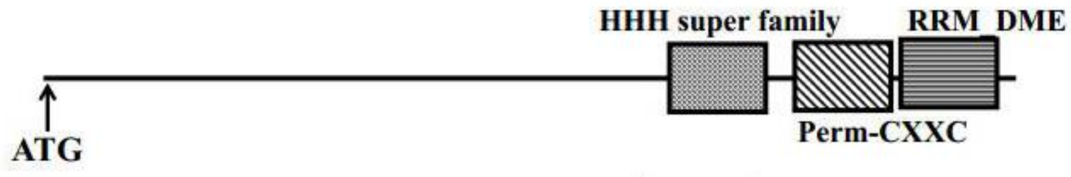

Supplementary Fig. S4. Structural analysis of *MdROS1* conserved domains (CDs). There are three domains in the *MdROS1* CD area: a helix-hairpin-helix motif (HHH super family, 3769 bp-4285 bp), a permuted single zf-CXXC domain (perm-CXXC, 4957 bp-5052 bp), and a RNA recognition motif-DME domain (RRD-DME, 5059 bp-5365 bp).

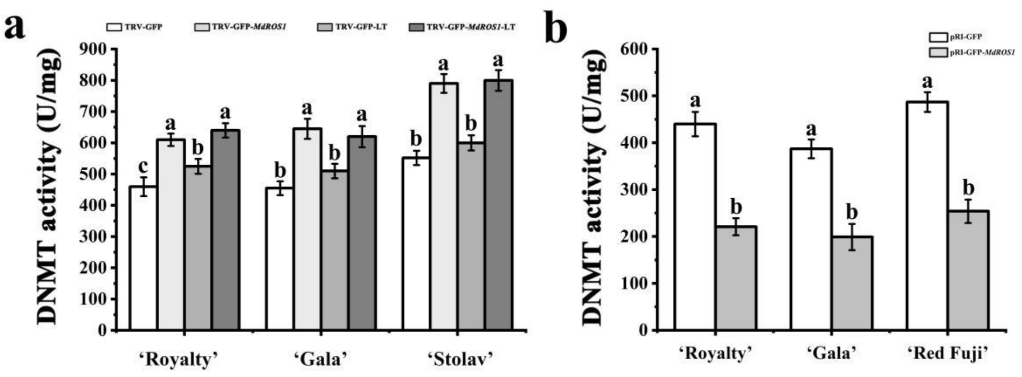

Supplementary Fig. S5. (a) The DNMT activity in *MdROS1* silenced leaves and fruits. (b) The DNMT activity in the *MdROS1*-overexpressed leaves and fruits. Different letters above the bars indicate significantly different values ($P < 0.05$) calculated using one-way analysis of variance (ANOVA) followed by a Tukey’s multiple range test.

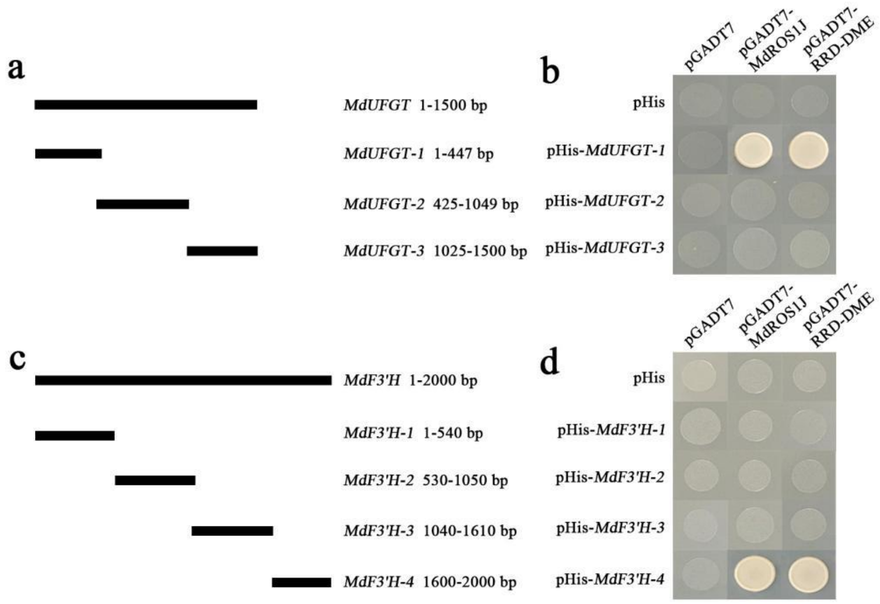

Supplementary Fig. S6. Yeast one-hybrid assay indicating that the RRD-DME domains and MdROS1J protein binds directly to the promoters of *MdF3'H-4* (1600 bp-2000 bp) and *MdUFGT-1* (1 bp-447 bp).
