# Supplementary material for: ROS1 promotes low temperature-induced anthocyanin accumulation in apple by demethylating the promoter of anthocyanin-associated genes
Source: Hortic Res. 2022 Feb 11;9:uhac007. doi: 10.1093/hr/uhac007 (PMC9123231; doi:10.1093/hr/uhac007)
Supplement: Web_Material_uhac007 [file web_material_uhac007.zip › Supplemetal Table.docx]

**Supplemental Tables**

Table S1 Primer sequences

| Primer | Sequences**（5’-3’)** | **Used for** |
| --- | --- | --- |
| MdROS1-1-F | \| ATGGGTGAACAGGGAGGAGAT \| \| --- \| | McROS1 CDs cloning |
| MdROS1-1-R | TTTTTGCTTGTTCTTCTATTGTC |  |
| MdROS1-2-F | TCAAAGAGAAATTATTCGAGTG |  |
| MdROS1-2-R | AGTTGCTCTCGCACGATCCTGT |  |
| MdROS1-3-F | AAACAGAGAATCCGCCTAATA |  |
| MdROS1-3-R | ATCTGGCTTGCTTCCAATTGCT |  |
| MdROS1-4-F | TGACGCAAAATCTGCAGTTGG |  |
| MdROS1-4-R | CTACTCCTCTCTCTTATCTTTT |  |
| HHH super family-F | GACTGGGAAGCCGTAAGATGTGCAG |  |
| HHH super family-R | GCCTACAATCTGTTCTCAGCGGGCA |  |
| RDD-DME-F | GGGACTCTCTTGATACCATGCCGAA |  |
| RDD-DME-R | CTGGGAAGTGCAGTCGTGCCATTAA |  |
| MdROS1J-F | GACTGGGAAGCCGTAAGATGTGCAG |  |
| MdROS1J-R | CTGGGAAGTGCAGTCGTGCCATTAA |  |
| MdCHS-Pro-F | CATGAATTTATGTCCTAATT | Promoter Cloning |
| MdCHS-Pro-R | TCCGATCGTCGAGAAAGATC |  |
| MdCHI-Pro-F | TGGCTCATGTGGGACACAAA |  |
| McCHI-Pro-R | TGTAGTGGAGGCGTGAAACC |  |
| MdF3’H-Pro-F | TGTGAACCTAGTCCCTTATT |  |
| MdF3’H-Pro-R | GCTGTTTAGATTGTGTGTGG |  |
| MdANS-Pro-F | GGATCCTAACATATGAATTTCGAAA |  |
| MdANS-Pro-R | TTTTGGAGCTGGCTTTCGACAACAA |  |
| MdUFGT-Pro-F | TACACAAATACATAGGTTCA |  |
| MdUFGT-Pro-R | TACAGCTTACAAGGCTAATT |  |
| MdMYB10-Pro-F | TCCAGGGGAGTGGATCTTGT |  |
| MdMYB10-Pro-R | GGATTCCGTTAAGCGGTCCA |  |
| qMd18s-F | GTCACTACCTCCCCGTGTCA | qRT-PCR |
| qMd18s-R | GAGCCTGAGAAACGGCTACC |  |
| qMdCHS-F | GGAGACAACTGGAGAAGGACTGGAA |  |
| qMdCHS-R | CGACATTGATACTGGTGTCTTCA |  |
| qMdCHI-F | GGGATAACCTCGCGGCCAAA |  |
| qMdCHI-R | GCATCCATGCCGGAAGCTACAA |  |
| qMdF3H-F | TGGAAGCTTGTGAGGACTGGGGT |  |
| qMdF3H-R | CTCCTCCGATGGCAAATCAAAGA |  |
| qMdF3’H-F | ACGATGGCGGATGTTACGG |  |
| qMcF3’H-R | GCTTTGACCCTGCACTTGCT |  |
| qMdDFR-F | GATAGGGTTTGAGTTCAAGTA |  |
| qMdDFR-R | TCTCCTCAGCAGCCTCAGTTTTCT |  |
| qMcANS-F | GAGAAGTATGCCAATGACCAGG |  |
| qMdANS-R | GGCGGTTGCCTCAATGTAAT |  |
| qMdUFGT-F | CCACCGCCCTTCCAAACACTCT |  |
| qMdUFGT-R | CACCCTTATGTTACGCGGCATGT |  |
| qMdMYB10-F | TGCCTGGACTCGAGAGGAAGACA |  |
| qMdMYB10-R | CCTGTTTCCCAAAAGCCTGTGAA |  |
| qMdROS1-F | CTCGGGAGCACCCATATGTC |  |
| qMdROS1-R | GGAAGGCTCAAGGGATGCTT |  |
| MdCHS-BP-F | TTGATTGGAAGTTATAGAATAGGGTTTT | Bisulfite Sequencing |
| MdCHS-BP-R | ATACCTTCACATAACCCAACAACAATCT |  |
| MdCHI-BP-F | TTTTATTTTTGGATGGTTATTTTTGTAGT |  |
| MdCHI-BP-R | CTCAATTTTCATACCTCCACTTTTTCA |  |
| MdF3’H-BP-F | TGTGAATTTAGTTTTTTATTGAATCGGA |  |
| MdF3’H-BP-R | AACCTTTTTATTTTAACAATATTATC |  |
| MdANS-BP-F | AATATAATATTTAGAGTTAATA |  |
| MdANS-BP-R | CATAATCCAAATTTTAACTATATA |  |
| MdUFGT-BP-F | TGATTAATTGGGAAGTTTTTATTTGAGTT |  |
| MdUFGT-BP-R | CTAAACTAAAATAAACAAAACTTCATATA |  |
| MdMYB10-BP-F | GTTAGTTTGTAATAGATTGAGATAGGT |  |
| MdMYB10-BP-R | AACAATTAAATTTCAAATAAAAAACTAC |  |
| pAD-HHH super family-F | GATGTGCCAGATTATGCCTCTCCCG GACTGGGAAGCCGTAAGATGTGCAG | Vectors construction |
| pAD-HHH super family-R | GCGAAGAAGTCCAAAGCTTCTCGAG GCCTACAATCTGTTCTCAGCGGGCA |  |
| pAD-RDD-DME-F | GATGTGCCAGATTATGCCTCTCCCG GGGACTCTCTTGATACCATGCCGAA |  |
| pAD-RDD-DME-R | GCGAAGAAGTCCAAAGCTTCTCGAG CTGGGAAGTGCAGTCGTGCCATTAA |  |
| pAD-MdROS1J-F | GATGTGCCAGATTATGCCTCTCCCG GACTGGGAAGCCGTAAGATGTGCAG |  |
| pAD-MdROS1J-R | GCGAAGAAGTCCAAAGCTTCTCGAG CTGGGAAGTGCAGTCGTGCCATTAA |  |
| pBD-MdCHS-Pro-F | TTCCTTTGATATTGGATCGGAATTC  CATGAATTTATGTCCTAATT |  |
| pBD-MdCHS-Pro-R | TATACATACAGAGCACATGCCTCGAG  TCCGATCGTCGAGAAAGATC |  |
| pBD-MdCHI-Pro-F | TTCCTTTGATATTGGATCGGAATTC  TGGCTCATGTGGGACACAAA |  |
| pBD-MdCHI-Pro-R | TATACATACAGAGCACATGCCTCGAG  TGTAGTGGAGGCGTGAAACC |  |
| pBD-MdF3’H-Pro-F | TTCCTTTGATATTGGATCGGAATTC  TGTGAACCTAGTCCCTTATT |  |
| pBD-MdF3’H-Pro-R | TATACATACAGAGCACATGCCTCGAG  GCTGTTTAGATTGTGTGTGG |  |
| pBD-MdANS-Pro-F | TTCCTTTGATATTGGATCGGAATTC  GGATCCTAACATATGAATTTCGAAA |  |
| pBD-MdANS-Pro-R | TATACATACAGAGCACATGCCTCGAG  TTTTGGAGCTGGCTTTCGACAACAA |  |
| pBD-MdUFGT-Pro-F | TTCCTTTGATATTGGATCGGAATTC  TACACAAATACATAGGTTCA |  |
| pBD-MdUFGT-Pro-R | TATACATACAGAGCACATGCCTCGAG  TACAGCTTACAAGGCTAATT |  |
| pBD-MdMYB10-Pro-F | TTCCTTTGATATTGGATCGGAATTC  TCCAGGGGAGTGGATCTTGT |  |
| pBD-MdMYB10-Pro-R | TATACATACAGAGCACATGCCTCGAG  GGATTCCGTTAAGCGGTCCA |  |
| pTRV2-GFP-MdROS1-F | GAGTAAGGTTACCGAATTCTCTAGAATGG  GTGAACAGGGAGGAGA |  |
| pTRV2-GFP-MdROS1-R | CCTCGAGACGCGTGAGCTCGGTACC  TCTTTTTGGTCTATTCAGG |  |
| pRI101-GFP-MdROS1-F | GTTCTTCACTGTTGATACAT  GGGACTCTCTTGATACCATG |  |
| pRI101-GFP-MdROS1-R | TCGCCCTTGCTCACCATGGA  CTGGGAAGTGCAGTCGTGCC |  |
| pHIS2-MdF3'H-1-F | TGTAATACGACTCACTATAGGGCG  CTGATGAAAAAAAAACAGTG |  |
| pHIS2-MdF3'H-1-R | GATCGATTCGCGAACGCGTGAGCT  AAATAAGCATGTTTATTAAT |  |
| pHIS2-MdF3'H-2-F | TGTAATACGACTCACTATAGGGCG  CTATTGATAACACATCATTT |  |
| pHIS2-MdF3'H-2-R | TGTAATACGACTCACTATAGGGCG  TATTGAATTATCTTTTATTC |  |
| pHIS2-MdF3'H-3-F | TGTAATACGACTCACTATAGGGCG  CTATTTCTAACGAAACTATC |  |
| pHIS2-MdF3'H-3-R | TGTAATACGACTCACTATAGGGCG  TTGTGCTAATGTGACTGAGA |  |
| pHIS2-MdF3'H-4-F | TGTAATACGACTCACTATAGGGCG  TCTCAGTCACATTAGCACAA |  |
| pHIS2-MdF3'H-4-R | TGTAATACGACTCACTATAGGGCG  GTGTTAGACTACGAGTGAAG |  |
| pHIS2-MdUFGT-1-F | TGTAATACGACTCACTATAGGGCG  TCTAAGCTTCTAACTCATCGAC |  |
| pHIS2-MdUFGT-1-R | TGTAATACGACTCACTATAGGGCG  CATTCTCCCACAATAATCTCTA |  |
| pHIS2-MdUFGT-2-F | TGTAATACGACTCACTATAGGGCG  ATTAGAGATTATTGTGGGAGAA |  |
| pHIS2-MdUFGT-2-R | TGTAATACGACTCACTATAGGGCG  ACATCACCCCCTTTCCGTTTAT |  |
| pHIS2-MdUFGT-3-F | TGTAATACGACTCACTATAGGGCG  AAACGGAAAGGGGGTGATGTGC |  |
| pHIS2-MdUFGT-3-R | TGTAATACGACTCACTATAGGGCG  TACAGCTTACAAGGCTAATTAG |  |
| pGADT7-ROSIJ-F | CATATGGCCATGGAGGCCAGTGAA  GACTGGGAAGCCGTAAGATGTGCAG |  |
| pGADT7-ROSIJ-R | CATATGGCCATGGAGGCCAGTGAA  CTGGGAAGTGCAGTCGTGCCATTAA |  |
| pGADT7-RRD-DME-F | CATATGGCCATGGAGGCCAGTGAA  GGGACTCTCTTGATACCATGCCGAA |  |
| pGADT7-RRD-DME-R | CATATGGCCATGGAGGCCAGTGAA  CTGGGAAGTGCAGTCGTGCCATTAA |  |
| M13-F | GTAAAACGACGGCCAGTGAATTCG |  |
| M13-R | CAGGAAACAGCTATGACCATGATTACG |  |
| pJG4–5-F | CCAGCCTCTTGCTGAGTGGAGATG |  |
| pJG4–5-R | AAGCCGACAACCTTGATTGGAG |  |
| PLacZi-F | AGAAGAACGGCATAGTGCGT |  |
| PLacZi-R | GCTACAAAGGACCTAATG |  |
| TRV1-F | TTACAGGTTATTTGGGCTAG |  |
| TRV1-R | CCGGGTTCAATTCCTTATC |  |
| TRV2-F | TGGGAGATGATACGCTGTT |  |
| TRV2-R | CCTAAAACTTCAGACACG |  |
| pBI121-McUFGT-Pro-F | AACAGCTATGACCATGATTACGCCACCACC  GCCCTTCCAAACACTCT |  |
| pBI121-McUFGT-Pro-R | AAGGGACTGACCACCCGGGGATCCTCA  CCCTTATGTTACGCGGCATGT |  |
| pBI121-McF3’H-Pro-F | AACAGCTATGACCATGATTACGCCACCACC  TGTGAACCTAGTCCCTTATT |  |
| pBI121-McF3’H-Pro-R | AAGGGACTGACCACCCGGGGATCCTCA  GCTGTTTAGATTGTGTGTGG |  |
