# Supplementary material for: ROS1 promotes low temperature-induced anthocyanin accumulation in apple by demethylating the promoter of anthocyanin-associated genes
Source: Hortic Res. 2022 Feb 11;9:uhac007. doi: 10.1093/hr/uhac007 (PMC9123231; doi:10.1093/hr/uhac007)
Supplement: Web_Material_uhac007 [file web_material_uhac007.zip › Supplemetal Table.pdf]

Supplemental Tables

Table S1 Primer sequences

Primer	Sequences（**5’-3’**)	**Used for**
MdROS1-1-F	ATGGGTGAACAGGGAGGAGAT	
MdROS1-1-R	TTTTTGCTTGTTCTTCTATTGTC	
MdROS1-2-F	TCAAAGAGAAATTATTCGAGTG	
MdROS1-2-R	AGTTGCTCTCGCACGATCCTGT	
MdROS1-3-F	AAACAGAGAATCCGCCTAATA	
MdROS1-3-R	ATCTGGCTTGCTTCCAATTGCT	
MdROS1-4-F	TGACGCAAAATCTGCAGTTGG	McROS1 CDs cloning
MdROS1-4-R	CTACTCCTCTCTCTTATCTTTT	
HHH super family-F	GACTGGGAAGCCGTAAGATGTGCAG	
HHH super family-R	GCCTACAATCTGTTCTCAGCGGGCA	
RDD-DME-F	GGGACTCTCTTGATACCATGCCGAA	
RDD-DME-R	CTGGGAAGTGCAGTCGTGCCATTAA	
MdROS1J-F	GACTGGGAAGCCGTAAGATGTGCAG	
MdROS1J-R	CTGGGAAGTGCAGTCGTGCCATTAA	
MdCHS-Pro-F	CATGAATTTATGTCCTAATT	
MdCHS-Pro-R	TCCGATCGTCGAGAAAGATC	
MdCHI-Pro-F	TGGCTCATGTGGGACACAAA	
McCHI-Pro-R	TGTAGTGGAGGCGTGAAACC	
MdF3’H-Pro-F	TGTGAACCTAGTCCCTTATT	
MdF3’H-Pro-R	GCTGTTTAGATTGTGTGTGG	Promoter Cloning
MdANS-Pro-F	GGATCCTAACATATGAATTTCGAAA	
MdANS-Pro-R	TTTTGGAGCTGGCTTTCGACAACAA	
MdUFGT-Pro-F	TACACAAATACATAGGTTCA	
MdUFGT-Pro-R	TACAGCTTACAAGGCTAATT	
MdMYB10-Pro-F	TCCAGGGGAGTGGATCTTGT	
MdMYB10-Pro-R	GGATTCCGTTAAGCGGTCCA	
qMd18s-F	GTCACTACCTCCCCGTGTCA	
qMd18s-R	GAGCCTGAGAAACGGCTACC	
qMdCHS-F	GGAGACAACTGGAGAAGGACTGGAA	
qMdCHS-R	CGACATTGATACTGGTGTCTTCA	
qMdCHI-F	GGGATAACCTCGCGGCCAAA	
qMdCHI-R	GCATCCATGCCGGAAGCTACAA	
qMdF3H-F	TGGAAGCTTGTGAGGACTGGGGT	
qMdF3H-R	CTCCTCCGATGGCAAATCAAAGA	qRT-PCR
qMdF3’H-F	ACGATGGCGGATGTTACGG	
qMcF3’H-R	GCTTTGACCCTGCACTTGCT	
qMdDFR-F	GATAGGGTTTGAGTTCAAGTA	
qMdDFR-R	TCTCCTCAGCAGCCTCAGTTTTCT	
qMcANS-F	GAGAAGTATGCCAATGACCAGG	
qMdANS-R	GGCGGTTGCCTCAATGTAAT	

qMdUFGT-F	CCACCGCCCTTCCAAACACTCT	
qMdUFGT-R	CACCCTTATGTTACGCGGCATGT	
qMdMYB10-F	TGCCTGGACTCGAGAGGAAGACA	
qMdMYB10-R	CCTGTTTCCCAAAAGCCTGTGAA	
qMdROS1-F	CTCGGGAGCACCCATATGTC	
qMdROS1-R	GGAAGGCTCAAGGGATGCTT	
MdCHS-BP-F	TTGATTGGAAGTTATAGAATAGGGTTTT	Bisulfite Sequencing
MdCHS-BP-R	ATACCTTCACATAACCCAACAACAATCT	
MdCHI-BP-F	TTTTATTTTTGGATGGTTATTTTTGTAGT	
MdCHI-BP-R	CTCAATTTTCATACCTCCACTTTTTCA	
MdF3’H-BP-F	TGTGAATTTAGTTTTTTATTGAATCGGA	
MdF3’H-BP-R	AACCTTTTTATTTTAACAATATTATC	
MdANS-BP-F	AATATAATATTTAGAGTTAATA	
MdANS-BP-R	CATAATCCAAATTTTAACTATATA	
MdUFGT-BP-F	TGATTAATTGGGAAGTTTTTATTTGAGTT	
MdUFGT-BP-R	CTAAACTAAAATAAACAAAACTTCATATA	
MdMYB10-BP-F	GTTAGTTTGTAATAGATTGAGATAGGT	
MdMYB10-BP-R	AACAATTAAATTTCAAATAAAAAACTAC	
pAD-HHH super family-F	GATGTGCCAGATTATGCCTCTCCCG<br>GACTGGGAAGCCGTAAGATGTGCAG	
pAD-HHH super family-R	GCGAAGAAGTCCAAAGCTTCTCGAG<br>GCCTACAATCTGTTCTCAGCGGGCA	
pAD-RDD-DME-F	GATGTGCCAGATTATGCCTCTCCCG<br>GGGACTCTCTTGATACCATGCCGAA	
pAD-RDD-DME-R	GCGAAGAAGTCCAAAGCTTCTCGAG<br>CTGGGAAGTGCAGTCGTGCCATTAA	
pAD-MdROS1J-F	GATGTGCCAGATTATGCCTCTCCCG<br>GACTGGGAAGCCGTAAGATGTGCAG	
pAD-MdROS1J-R	GCGAAGAAGTCCAAAGCTTCTCGAG<br>CTGGGAAGTGCAGTCGTGCCATTAA	
pBD-MdCHS-Pro-F	TTCCTTTGATATTGGATCGGAATTC<br>CATGAATTTATGTCCTAATT	Vectors construction
pBD-MdCHS-Pro-R	TATACATACAGAGCACATGCCTCGAG<br>TCCGATCGTCGAGAAAGATC	
pBD-MdCHI-Pro-F	TTCCTTTGATATTGGATCGGAATTC<br>TGGCTCATGTGGGACACAAA	
pBD-MdCHI-Pro-R	TATACATACAGAGCACATGCCTCGAG<br>TGTAGTGGAGGCGTGAAACC	
pBD-MdF3’H-Pro-F	TTCCTTTGATATTGGATCGGAATTC<br>TGTGAACCTAGTCCCTTATT	
pBD-MdF3’H-Pro-R	TATACATACAGAGCACATGCCTCGAG<br>GCTGTTTAGATTGTGTGTGG	
pBD-MdANS-Pro-F	TTCCTTTGATATTGGATCGGAATTC<br>GGATCCTAACATATGAATTTCGAAA	

pBD-MdANS-Pro-R	TATACATACAGAGCACATGCCTCGAG<br>TTTTGGAGCTGGCTTTCGACAACAA
pBD-MdUFGT-Pro-F	TTCCTTTGATATTGGATCGGAATTC<br>TACACAAATACATAGGTTCA
pBD-MdUFGT-Pro-R	TATACATACAGAGCACATGCCTCGAG<br>TACAGCTTACAAGGCTAATT
pBD-MdMYB10-Pro-F	TTCCTTTGATATTGGATCGGAATTC<br>TCCAGGGGAGTGGATCTTGT
pBD-MdMYB10-Pro-R	TATACATACAGAGCACATGCCTCGAG<br>GGATTCCGTTAAGCGGTCCA
pTRV2-GFP-MdROS1-F	GAGTAAGGTTACCGAATTCTCTAGAATGG<br>GTGAACAGGGAGGAGA
pTRV2-GFP-MdROS1-R	CCTCGAGACGCGTGAGCTCGGTACC<br>TCTTTTTGGTCTATTCAGG
pRI101-GFP-MdROS1-F	GTTCTTCACTGTTGATACAT<br>GGGACTCTCTTGATACCATG
pRI101-GFP-MdROS1-R	TCGCCCTTGCTCACCATGGA<br>CTGGGAAGTGCAGTCGTGCC
pHIS2-MdF3'H-1-F	TGTAATACGACTCACTATAGGGCG<br>CTGATGAAAAAAAAACAGTG
pHIS2-MdF3'H-1-R	GATCGATTCGCGAACGCGTGAGCT<br>AAATAAGCATGTTTATTAAT
pHIS2-MdF3'H-2-F	TGTAATACGACTCACTATAGGGCG<br>CTATTGATAACACATCATTT
pHIS2-MdF3'H-2-R	TGTAATACGACTCACTATAGGGCG<br>TATTGAATTATCTTTTATTC
pHIS2-MdF3'H-3-F	TGTAATACGACTCACTATAGGGCG<br>CTATTTCTAACGAAACTATC
pHIS2-MdF3'H-3-R	TGTAATACGACTCACTATAGGGCG<br>TTGTGCTAATGTGACTGAGA
pHIS2-MdF3'H-4-F	TGTAATACGACTCACTATAGGGCG<br>TCTCAGTCACATTAGCACAA
pHIS2-MdF3'H-4-R	TGTAATACGACTCACTATAGGGCG<br>GTGTTAGACTACGAGTGAAG
pHIS2-MdUFGT-1-F	TGTAATACGACTCACTATAGGGCG<br>TCTAAGCTTCTAACTCATCGAC
pHIS2-MdUFGT-1-R	TGTAATACGACTCACTATAGGGCG<br>CATTCTCCCACAATAATCTCTA
pHIS2-MdUFGT-2-F	TGTAATACGACTCACTATAGGGCG<br>ATTAGAGATTATTGTGGGAGAA
pHIS2-MdUFGT-2-R	TGTAATACGACTCACTATAGGGCG<br>ACATCACCCCCTTTCCGTTTAT
pHIS2-MdUFGT-3-F	TGTAATACGACTCACTATAGGGCG<br>AAACGGAAAGGGGGTGATGTGC

pHIS2-MdUFGT-3-R	TGTAATACGACTCACTATAGGGCG<br>TACAGCTTACAAGGCTAATTAG
pGADT7-ROSIJ-F	CATATGGCCATGGAGGCCAGTGAA<br>GACTGGGAAGCCGTAAGATGTGCAG
pGADT7-ROSIJ-R	CATATGGCCATGGAGGCCAGTGAA<br>CTGGGAAGTGCAGTCGTGCCATTAA
pGADT7-RRD-DME-F	CATATGGCCATGGAGGCCAGTGAA<br>GGGACTCTCTTGATACCATGCCGAA
pGADT7-RRD-DME-R	CATATGGCCATGGAGGCCAGTGAA<br>CTGGGAAGTGCAGTCGTGCCATTAA
M13-F	GTAAAACGACGGCCAGTGAATTCG
M13-R	CAGGAAACAGCTATGACCATGATTACG
pJG4–5-F	CCAGCCTCTTGCTGAGTGGAGATG
pJG4–5-R	AAGCCGACAACCTTGATTGGAG
PLacZi-F	AGAAGAACGGCATAGTGCGT
PLacZi-R	GCTACAAAGGACCTAATG
TRV1-F	TTACAGGTTATTTGGGCTAG
TRV1-R	CCGGGTTCAATTCCTTATC
TRV2-F	TGGGAGATGATACGCTGTT
TRV2-R	CCTAAAACTTCAGACACG
pBI121-McUFGT-Pro-F	AACAGCTATGACCATGATTACGCCACCACC<br>GCCCTTCCAAACACTCT
pBI121-McUFGT-Pro-R	AAGGGACTGACCACCCGGGGATCCTCA<br>CCCTTATGTTACGCGGCATGT
pBI121-McF3’H-Pro-F	AACAGCTATGACCATGATTACGCCACCACC<br>TGTGAACCTAGTCCCTTATT
pBI121-McF3’H-Pro-R	AAGGGACTGACCACCCGGGGATCCTCA<br>GCTGTTTAGATTGTGTGTGG
